# Identifying Common Patient‐Oriented Priorities for Child and Adolescent Health Research and Care: A Systematic Review of Priority Setting Partnerships

**DOI:** 10.1111/hex.70349

**Published:** 2025-07-30

**Authors:** Jenna S. Jessa, Muning (Linda) Zhang, Justin Bonhomme, Dawn P. Richards, Diane L. Lorenzetti, Christine T. Chambers, Kathryn A. Birnie

**Affiliations:** ^1^ Department of Anesthesiology, Perioperative & Pain Medicine University of Calgary Calgary Canada; ^2^ Cumming School of Medicine University of Calgary Calgary Canada; ^3^ Alberta Children's Hospital Research Institute Calgary Canada; ^4^ Faculty of Occupation Science University of Western Ontario London Canada; ^5^ Five02 Labs Inc Toronto Canada; ^6^ Health Sciences Library University of Calgary Calgary Canada; ^7^ Department of Community Health Sciences University of Calgary Calgary Canada; ^8^ Department of Psychology and Neuroscience Dalhousie University Halifax Canada; ^9^ Department of Pediatrics Dalhousie University Halifax Canada

**Keywords:** adolescent, child, health policy, health research, James Lind Alliance, patient engagement, priority setting

## Abstract

**Context:**

James Lind Alliance (JLA) Priority Setting Partnerships (PSPs) bring together patients, clinicians and carers (e.g., parents, guardians or family members) to identify the most important questions in specific areas of health research. The inclusion of diverse perspectives in PSPs can inform priority‐guided investment in child health.

**Objective:**

The objective of this study is to explore top priority areas identified in JLA PSPs across a range of child health conditions.

**Evidence Review:**

This systematic review was designed to capture JLA PSPs conducted on health topics relevant to children and youth. Database searches were run in MEDLINE, CINAHL, Embase and APA PsycINFO, and a grey literature search was conducted on the JLA's official website for reports published up to 1 August 2023. English‐language articles adhering to, or adapting from, the JLA PSP methodology were eligible for inclusion if they focused on child and youth health, development and well‐being. A thematic synthesis was conducted to explore common themes amongst all the top 10 lists.

**Main Results:**

The systematic review identified 42 unique PSPs focused on child and youth health published from 2010 onwards. A total of 578 priorities were analysed across the top 10 priority lists. While the identified PSPs were conducted over a broad range of child health topic areas (e.g., general well‐being, mental health, neurodevelopmental, and acute and chronic conditions), 18 common themes were identified across the top 10 priority lists.

**Conclusions and Relevance:**

Our findings provide an overview of the JLA PSP process and common areas of interest in child health research. This study can guide health research funding, health system responsiveness and policymakers' actions for widescale improvement in child and youth health.

**Patient or Public Contribution:**

A person with lived experience who is also an expert in patient partnership and has been involved in multiple JLA PSPs was engaged as a compensated member of the project team. They were involved in the initial idea for the review, the design of the review question and methodology, the analysis and interpretation of the review findings, and co‐authorship of the manuscript.

## 1 Introduction

Despite improved child mortality over the past 30 years, the global burden of disease in children remains high, with increased overall morbidity [1, 2]. Continued priority‐driven investment in child health, including for non‐fatal illness and disability, is critically needed to address top risk factors and ensure optimal development and well‐being for all children [3]. In 2015, all Member States of the United Nations adopted the Sustainable Development Goals (SDGs), 17 shared global goals that form a blueprint to end poverty, improve health and education, reduce inequality, and build more peaceful, prosperous societies by 2030 [4]. As underscored by UNICEF, significant accelerated effort is required if we are to reach these global goals for children, including a global shift from treating diseases to strengthening health systems [4, 5].

Inclusive and equitable engagement with children and their families is one valuable approach to achieving transformative change in child health and well‐being. The 1989 Convention on the Rights of the Child recognises that children have their own voice, agency and right to participate in decisions that affect them [6]. The involvement of patients and members of the public in research activities, including in research priority setting, conduct and governance, has also been increasingly identified as critical to ensuring research relevance, effectiveness and impact [7, 8]. Indeed, research priorities differ when people with lived experience and clinicians are engaged versus researchers alone [9, 10], underscoring the importance of engaging children and their families in efforts to identify what areas are most critical for action. Identifying and addressing patient needs could contribute to more efficient clinical research and yield clinical and cost‐effective benefits by cultivating guided and relevant research directions [10].

Established in 2004, the James Lind Alliance (JLA) is a non‐profit initiative designed to bring patients, clinicians and carers (e.g., parents, caregivers and family members) together in Priority Setting Partnerships (PSPs) to identify the most important questions for health research [11]. As stated by the JLA founders, the involvement of patients in research priority setting was intended to ensure that: ‘important questions are not overlooked because of emphasis on: chronic but not acute health problems; severe but not common health problems; and disease‐specific but not crosscutting issues’ [12]. JLA PSPs stand out from other priority‐setting processes in health research given their recognised approach to prioritising the non‐researcher voice [13], as well as for their robust, inclusive and objectively based approach applicable across health conditions [14]. The strategic steps to a JLA PSP are an in‐depth and established process involving the formation of a steering committee, rounds of surveys to gather initial questions and rate their importance, a comparison to existing knowledge, a workshop to reach consensus on the final top priorities, and publication of a final top 10 priorities list [15]. Furthermore, patient partnership and engagement, as undertaken in JLA PSPs, is recognised as important in the move towards greater quality, accountability and accessibility in healthcare [14, 16].

JLA PSPs have covered broad health topics and populations, including child health [11, 17]. Within child health topics, the identified top 10 patient priorities overlap with SDGs identified by UNICEF as critical to improving health, including access to essential health services, vaccine adherence and obesity incidence [4]. Common priorities identified across child health‐related JLA PSPs, identified in partnership with children and their families, may better inform research and clinical practice, thereby meaningfully addressing diverse health conditions affecting children. As such, we conducted a systematic review to determine the top shared priority areas across JLA PSPs related to child health.

## 2 Methods

This review followed PRISMA reporting guidelines for quality reporting of systematic reviews [18]. The search strategy was developed with a medical librarian and incorporated terms from a paediatric search filter [19] (see Supporting Information S1 for complete search strategy). Searches were conducted on 1 August 2023 in Medline, CINAHL, Embase and PsycINFO databases and PSP Top 10 priority lists published on the JLA website [20] (p1). The goal was to capture all JLA PSPs conducted on topics relevant to children and adolescents. Articles were included that: (1) identified the method used as a JLA PSP, or an adapted JLA PSP that maintained methodological principles of the PSP method (e.g., balanced inclusion of patient, caregiver and health professional voices; initial, interim and final priority‐setting workshop; final list of top priorities); (2) related to child and adolescent health, development and/or well‐being; (3) were a completed PSP; and (4) published in English. Exclusion criteria included: (1) studies focusing on infant or maternal health and (2) research letters, secondary analyses, editorials and opinion pieces.

An additional grey literature search was conducted by searching PSPs published on the JLA website [17]. As required for data extraction, PSPs were included if there was an available report or publication outlining the process and/or details about the project itself, as well as a final top 10 priority list. Topics relevant to child and youth populations were searched first, followed by an examination of the topics that seemed broadly applicable to more populations. Topics not relevant to child and youth populations (e.g., seniors' health) were not searched. The results of the grey literature search and database searches were cross‐referenced and compared to identify duplicate PSPs. When both a published article and a report were available, data were collected from the published article.

Abstracts and full‐text articles were screened by a primary reviewer. A random 20% of abstracts and full‐text articles were independently screened by a secondary reviewer to demonstrate excellent interrater reliability (89.2% and 86.4%, respectively). Extracted data were informed by the Reporting guideline for priority setting of health research [21] and included context and scope, governance and team, participants, identification and collection of research priorities, prioritisation of research questions, output, evaluation and feedback, implementation/dissemination, funding and conflict of interest.

Data were analysed using thematic synthesis [4] to identify commonalities between the final eligible JLA PSP top 10 research priority lists (see Table S1 for thematic synthesis). As per thematic synthesis, two coders, in collaboration with co‐authors, coded text and developed descriptive themes that preserve the link between conclusions and the text of primary studies [22]. No quality assessment method has been established to evaluate JLA PSPs; therefore, none was included in this review.

## 3 Results

### 3.1 Study Selection

**Figure 1:**
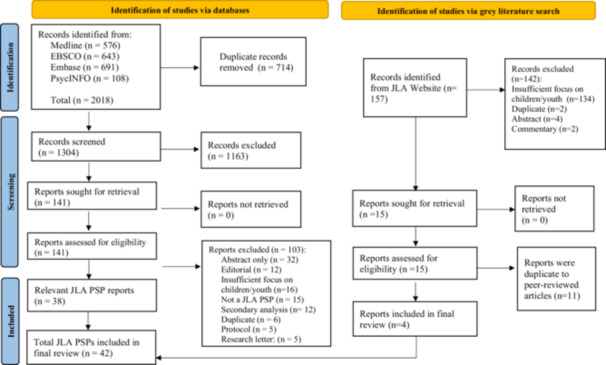
PRISMA flow diagram.

A total of 42 JLA PSPs were identified from scientific and grey literature searches and included in this systematic review (see Table 1). The scientific database searches identified a total of 1148 unique abstracts screened for eligibility. Of these, 203 (17.7%) were independently screened by two reviewers with an interrater agreement of 89.2%. A total of 141 full‐text articles were screened, with 22 (15.6%) independently screened by two reviewers with an interrater agreement of 86.4%. All conflicts were discussed and resolved by a third reviewer. A total of 38 scientific articles were included in the final data extraction. The grey literature search on the JLA website was conducted by one author and yielded 15 PSPs that met the inclusion criteria. Each PSP from the JLA website was reviewed and cross‐referenced with the results of the scientific database searches and yielded an additional four PSPs for inclusion. See Figure 1 for the PRISMA flow diagram.

**Table 1:** Included JLA PSP characteristics.

Year of publication	Frequency (%)	
2010	2 (4.8%)	
2011	0 (0%)	
2012	1 (2.4%)	
2013	2 (4.8%)	
2014	1 (2.4%)	
2015	2 (4.8%)	
2016	2 (4.8%)	
2017	2 (4.8%)	
2018	5 (11.9%)	
2019	6 (14.3%)	
2020	4 (9.5%)	
2021	4 (9.5%)	
2022	7 (16.7%)	
2023	4 (9.5%)	
Population/Topic of focus		
Mental health	3 (7.1%)	
Anaesthesia	2 (4.8%)	
General child health	2 (4.8%)	
Juvenile idiopathic arthritis	2 (4.8%)	
Psoriasis	2 (4.8%)	
Acne	1 (2.4%)	
Anorexia nervosa	1 (2.4%)	
Asthma	1 (2.4%)	
Attention deficit/hyperactivity disorder	1 (2.4%)	
Cancer	1 (2.4%)	
Childhood neurological disorders	1 (2.4%)	
Children and young people with neurodisability	1 (2.4%)	
Children requiring elective surgery for conditions affecting the lower limbs	1 (2.4%)	
Children with Down Syndrome, cleft lip with or without cleft palate, congenital heart defects or spina bifida	1 (2.4%)	
Chronic conditions and disabilities	1 (2.4%)	
Congenital heart disease	1 (2.4%)	
Cystic fibrosis	1 (2.4%)	
Dysphagia	1 (2.4%)	
Dystonia in cerebral palsy patients	1 (2.4%)	
Dystrophic epidermolysis bullosa	1 (2.4%)	
Eczema	1 (2.4%)	
Hyperacusis	1 (2.4%)	
Inflammatory bowel disease	1 (2.4%)	
Kidney transplant	1 (2.4%)	
Learning difficulties	1 (2.4%)	
Lichen sclerosus	1 (2.4%)	
Liver glycogen storage diseases	1 (2.4%)	
Paediatric and child health nursing	1 (2.4%)	
Paediatric chronic pain	1 (2.4%)	
Paediatric hospital inpatients	1 (2.4%)	
Paediatric inflammatory bowel disease	1 (2.4%)	
Paediatric preventative care research	1 (2.4%)	
PICU nutrition	1 (2.4%)	
Retinoblastoma	1 (2.4%)	
Sight loss and vision	1 (2.4%)	
Urinary incontinence	1 (2.4%)	
Country		
The United Kingdom	22 (50%)	
Canada	8 (18.2)	
International	4 (9.5%)	
Australia	3 (6.8%)	
The Netherlands	2 (4.5%)	
The United States	1 (2.3%)	
Sweden	1 (2.3%)	
Spain	1 (2.3%)	
Type of funder		
Research network	16 (30.8%)	
Charitable organisations	14 (26.9%)	
Other research funds (e.g., university grant and other grants)	12 (23.1%)	
Tri‐Council (CIHR, SSHRC, and NSERC)**	6 (11.5%)	
Not‐for‐profit organisations	4 (7.7%)	

Sample size	Median [IQR]	Range	
Sample size		
Initial survey*	338 [142–546]	14–2566	
Interim survey*	250 [82–615]	12–2822	
Final workshop*	22 [19–28]	5–155	
Number of uncertainties		
Initial uncertainties	472 [108–1065]	10–8276	
Final uncertainties	10 [10–10]	4–27	

** Refers to the national Tri‐Council funding agencies in Canada, including the Canadian Institutes of Health Research (CIHR), the Social Sciences and Humanities Research Council (SSHRC) and the Natural Sciences and Engineering Research Council (NSERC).

* Different participant groups (i.e., children/adolescents/people with lived experience, parents/caregivers/family members, clinicians/health professionals and others) collapsed into overall sample size for each stage.

#### 3.1.1 Study Characteristics

Table 1 provides an overview of the population and setting of the included JLA PSPs (see Table S2 for characteristics about individual included JLA PSPs). The earliest study was published in June 2010 (see Figure S1). Close to half of the studies (48%, *n* = 20) were conducted in or based out of the United Kingdom, followed by Canada (19%, *n* = 8) and Australia (7%, *n* = 3). Four studies had international participation (10%, *n* = 4) and one study had cross‐Europe participation (2%, *n* = 1).

Of the 42 unique PSPs identified, 39 were published in peer‐reviewed journals (93%), and 3 were identified from reports on the JLA website with no corresponding peer‐reviewed publication [23, 24, 25]. The majority of identified JLA PSPs focused on specific diseases or conditions (81%, *n* = 35), and the others were about services, procedures or interventions (17%, *n* = 7).

#### 3.1.2 PSP Leaders and Funders

Most of the identified PSPs were led by academic organisations (50%, *n* = 21), followed by healthcare organisations (34%, *n* = 14), health charities (17%, *n* = 7), research networks (14%, *n* = 6) and not‐for‐profit organisations (7%, *n* = 3) as defined in included papers.

The majority of the PSPs were also done in partnership with another type of organisation (81%, *n* = 34), with the most common partnership occurring with not‐for‐profits and charitable organisations (57%, *n* = 24). Most of the identified PSPs received funding (81%, *n* = 34), usually from multiple sources. The most common source of funding came from research networks (40%, *n* = 17) and charitable or not‐for‐profit organisations (40%, *n* = 17), followed by national health research funding (14%, *n* = 6) and other research funding (e.g., start‐up funds, university grants and other research grants) (14%, *n* = 6).

#### 3.1.3 PSP Participant Involvement

Results are presented here in accordance with the core steps of JLA PSP methodology involving participant engagement, including: (1) initial surveys with patients, carers and clinicians to gather evidence uncertainties, (2) interim surveys with patients, carers and clinicians to shortlist evidence uncertainties, and (3) final consensus workshops to rank and order shortlisted evidence uncertainties to a final top 10 list [11].

##### 3.1.3.1 Initial Survey

There were 34 (81%) studies that included children/adolescents/people with lived experience in the initial survey, 37 (88%) studies which included parents/caregivers/family members and 38 (90%) studies that included clinicians/health professionals. The median number of participants across the initial survey stage was 312, with a range of 14–2566. Race and/or ethnicity was reported in 14 (34%) studies, and sex and/or gender was reported in 24 (57%) studies.

##### 3.1.3.2 Interim Survey

There were 33 (79%) studies which included children/adolescents/people with lived experience, and 36 (86%) studies that included parents/caregivers/family members in the interim survey rating/ranking the importance of potential priorities. Additionally, 37 (88%) studies included clinicians/health professionals. The median number of participants across the initial survey stage was 253, with a range of 14–2822. Race and/or ethnicity was reported in 11 (26%) studies, and sex and/or gender was reported in 20 (48%) studies.

##### 3.1.3.3 Final Consensus Workshop

There were 36 (86%) studies which included children/adolescents/people with lived experience, 30 (70%) studies that included parents/caregivers/family members, and 38 (90%) studies that included clinicians/health professionals in the final workshop to reach consensus on the top final priorities. The median number of participants in the final workshop stage was 22, with a range of 5–155 across 1–12 workshop sessions. Race and/or ethnicity were reported in 5 (12%) studies, and sex and/or gender were reported in 11 (26%) studies.

### 3.2 Final ‘Top 10’ Priority Lists

The majority of studies (*n* = 36; 86%) had 10 final research priorities, and 7 studies (17%) had a different number of final research priorities (top 4, 5, 6, 11, 14, 20 and 27). A total of 54 top priority lists were included, as some papers included separate priority lists for different diseases/populations [26, 27] and age categories [28, 29].

A total of 578 priorities across all 54 final priority lists were coded into 18 common themes. Table 2 provides an overview of thematic coding, including operational definitions for themes and numbers and percentages of all top priorities identified across included JLA PSPs. General symptom treatment and management, non‐pharmacological interventions, and biomedical interventions were the top three themes highlighted from all included JLA PSPs. Additional themes included non‐treatment‐focused priorities (i.e., screening, assessment and monitoring; prevention, prognosis and disease course; aetiology and risk factors; and family, caregiver and peer support) and systems‐level factors (i.e., public health, health professional training, integrated knowledge translation, and care coordination and health systems navigation). Priorities could be coded into multiple themes if relevant, which occurred for 34 priorities.

**Table 2:** Common themes across JLA PSP child and adolescent ‘top 10’ priorities.

Theme	Frequency	Example priorities
General symptom treatment and management	74 (12.8%)	1. What are the effective ways of simplifying the treatment burden of people with Cystic Fibrosis? 2. What management strategy should be adopted for the treatment of acne to optimise short‐ and long‐term outcomes?
General questions related to treatments that can improve or manage the symptoms or condition of patients with a disease. These include questions about treatment efficacy/effectiveness, dosage, safety, timing, long‐term risk and considerations for specific subpopulations.		
Non‐pharmacological interventions	69 (11.9%)	1. Do lifestyle factors such as diet. dietary supplements, alcohol, smoking, weight loss and exercise play a part in treating psoriasis? 2. What interventions, including self‐care, can reduce or reverse the adverse short‐term and long‐term effects of cancer treatment?
Questions related to the effectiveness of non‐pharmacological interventions for the prevention, treatment or management of a disease or disorder. Non‐pharmacological interventions include complementary and alternative therapies, psychological treatment, exercise‐based treatment and/or nutritional/dietary interventions.		
Biomedical interventions	61 (10.6%)	1. What are the adverse effects associated with long‐term use of short‐ and long‐acting bronchodilators; inhaled and oral steroids; and combination and additive therapies in children? 2. Is there a risk that medication with methylphenidate during childhood will lead to the development of drug dependence later in life?
Questions concerning the effectiveness, safety and evaluation of biomedical therapies for disease. These include pharmaceutical drugs, surgical and/or biologic therapy.		
Public health	48 (8.3%)	1. How can we raise awareness, increase community inclusion and reduce the exclusion and isolation of young people with chronic conditions and disabilities in all aspects of life? 2. What is the prevalence of hyperacusis in a general population and other specific populations (e.g., people with autism, mental health issues, learning disabilities and hearing loss)?
Questions related to public health, community health or health promotion. These include questions about social determinants of health, disease prevalence or behaviours that promote health and well‐being.		
Screening, assessment and monitoring	44 (7.6%)	1. What are the optimal markers/combinations of markers (clinical, endoscopic, imaging, genetics and other biomarkers) for stratification of patients with regard to (a) disease course, (b) monitoring disease activity and (c) treatment response? 2. How to increase early diagnosis of retinoblastoma (i.e., decrease age or stage at diagnosis)?
Questions concerning the screening, diagnosis, assessment and/or monitoring of a health condition.		
Prevention and early intervention	43 (7.4%)	1. How can cataract be prevented in children? 2. Can early therapy interventions improve functional and developmental outcomes in babies experiencing brain injury during pregnancy or infancy?
Questions concerning methods for preventing and/or slowing the development of disease.		
Prognosis and disease course	41 (7.1%)	1. What are the most helpful and least helpful treatment elements as identified by recovered individuals, and what long‐term outcomes do they perceive them to help with? 2. What treatments or strategies effectively prevent acute pain from becoming chronic in children and adolescents?
Questions concerning disease prognosis. These include questions about disease course and outcomes, as well as the effects of medication/treatments on prognosis.		
Potential interventions	39 (6.7%)	1. Can new non‐invasive treatments be developed to slow down the progression of diabetic retinopathy? 2. How can existing cornstarch preparations be modified or alternative treatments be implemented that are easier to administer and/or keep blood sugar levels more stable for patients with liver GSD?
Questions related to the development of new interventions or the improvement of existing interventions in the treatment of a health condition. These include questions about the therapeutic potential of newly emerging treatment technologies and/or potential applications of existing therapies in novel ways.		
Aetiology and risk factors	29 (5.0%)	1. What are the causes of IBD (Crohn's disease or ulcerative colitis)? 2. How can the risk of losing sight for people with retinal detachment be reduced?
Questions related to the underlying cause(s) of a disease, or its signs/symptoms, and factors that predict its development. These could include genetic and/or lifestyle factors.		
Mental health	25 (4.3%)	1. What are the best strategies for the prevention of mental health issues in children and families? 2. What psychological support package improves psychological well‐being, social functioning and mental health during and after treatment?
Questions related to the psychological, social or behavioural impacts of a disease, including but not limited to mental health conditions.		
Family, caregiver and peer support	20 (3.5%)	1. What methods of communication are most effective between patients, caregivers and healthcare providers on a GPIU? 2. How to provide culturally competent social, emotional and psychological support to patients with retinoblastoma, survivors, parents and families (at diagnosis and beyond)?
Questions concerning the role of family, caregiver and peer relationships in supporting health and well‐being, or in the treatment and management of a disease or disability. These include questions about shifting the responsibility of care to caregivers and available resources for caregivers to support the child's health and well‐being.		
Healthcare professional training	17 (2.9%)	1. What knowledge, skills and training do educational professionals need to identify the early signs of learning difficulties and provide optimal support for children and young people affected to help them achieve the best possible outcomes? 2. Can guidance or training for general practitioners on appropriate pathways of care improve the management of patients with urinary incontinence?
Questions concerning healthcare professional training. These include questions about improving healthcare professional training as well as the association between healthcare professional training and the care, support, management or referral of patients with a disease or disability.		
Comorbidities	14 (2.4%)	1. What is the most effective way of managing asthma with other health problems? 2. Does treating psoriasis help improve other health conditions, such as psoriatic arthritis, cardiovascular disease, metabolic syndrome and stress?
Questions concerning the risk of developing a comorbid health condition and/or the treatment or management of comorbidities.		
Early education	14 (2.4%)	1. What is the best educational and community environment for children and young people with learning difficulties? 2. How does children's educational experience impact their health and well‐being?
Questions concerning the relationship between early education and disease, health or child development.		
Quality of life	12 (2.1%)	1. What is the influence of JIA on future opportunities regarding school results, work and relationships? 2. What is the impact of living with CHD on the quality of life in children, and how can this be improved?
Questions concerning the impact of a disease, condition or disability on a patient's or family's quality of life and how quality of life can be improved.		
Integrated Knowledge Translation	11 (1.9%)	1. Can consensus guidelines (for management) be achieved for patients with liver GSD? 2. What are the impacts of involving patients in shared decision‐making about anaesthesia and care options before, during and after surgery?
Questions related to the utility of new data to increase understanding or improve the health and well‐being of patients. This includes questions about mobilising and implementing the latest research findings, as well as knowledge from patients, caregivers and healthcare professionals about a disease, condition or disability.		
Care coordination and health systems navigation	9 (1.6%)	1. What best practices and/or care models exist for inpatient care for children and youths with medical complexity on the GPIU? How can multiple types of professionals work together with parents and carers to improve identification, diagnosis, interventions and treatments and achieve the best outcomes for children and young people with learning difficulties?
Questions related to transitions, navigation and/or coordination of care within the healthcare system and at home.		
Treatment effect modifiers	9 (1.6%)	1. What factors predict how well psoriasis will respond to a treatment? Which factors before, during and after receiving anaesthesia for surgery are most important to improve patient outcomes and satisfaction?
Questions concerning the identification of patient characteristics or factors associated with treatment/intervention response.		

### 3.3 Dissemination of PSPs

26 (62%) of the studies in the article mentioned their plans to disseminate their findings after publication. Many mentioned partnering with project partners to distribute the findings, as well as ways of translating knowledge to make it available to all stakeholders, potential funding bodies and the general public. Despite the majority of studies indicating some direction for sharing their results, only 4 (9.5%) of the studies indicated how or when they would be evaluating the engagement of their study after publication.

## 4 Discussion

This systematic review is the first to provide a comprehensive overview and thematic analysis of top research priorities amongst JLA PSPs focused on child and adolescent health. In the spirit of the origins of the JLA PSPs [12], our goal with this review was to emphasise cross‐cutting versus disease‐specific issues to ensure that these are not overlooked in our collaborative, collective efforts to improve the health and well‐being of all children. In total, 42 articles were included (with 47 priority lists), with the first published in 2010, and the majority coming from research teams in the United Kingdom or Canada. Articles predominantly focused on specific disease populations, and most studies were conducted in partnership with JLA. While all included articles utilised the JLA PSP methodology, a minority used adapted methodology that largely included a modified review of existing literature opening the possibility that some identified research priorities may have already been addressed by existing research. The majority of PSPs focused on physical health conditions (e.g., arthritis and asthma), with a minority of PSPs centred on mental health. Across all PSPs, a total of 578 priorities were coded into 18 themes. The largest proportion of priorities related to general symptom treatment and management, followed by non‐pharmacological and then biomedical interventions; however, altogether these accounted for just over one‐third of all priorities. Many non‐treatment‐focused priority themes were identified such as screening, assessment and monitoring, prevention, prognosis and disease course, aetiology and risk factors, and family, caregiver and peer support, as well as themes addressing more systems‐level factors such as public health, health professional training, integrated knowledge translation, and care coordination and health systems navigation. Similar reviews of JLA PSPs in adult populations revealed that the majority of priorities were either broad research topics or followed the population‐intervention‐control‐outcome (PICO) framework [30] and that different priority themes emerged from PSPs from the United Kingdom (i.e., focused on interventions) versus from the United States (i.e., focused on predictive factors of health) [31].

Previous synthesis of JLA PSPs has demonstrated similar thematic analyses, with high‐level themes including quality of life, caregivers and families, causes and prevention, screening and diagnosis, treatment and management, services and systems, and social influences and impacts [32], all of which were identified in the present study. Given the limited synthesis of child‐specific JLA PSPs, we recognise that our thematic findings of prognosis and disease course, early education and integrated knowledge translation are novel amongst the landscape of existing reviews of PSPs. Given the lack of previous reports of these priority themes in adult populations, the need for specific focus on child and adolescent health is warranted, specifically given that prognosis and early education are specific to this population.

This review included priorities identified by people with lived experience, family members and health professionals in JLA PSPs across any area of child and adolescent health anywhere in the world. The United Nations' SDGs offer a shared blueprint to transform the lives of all people (including children and adolescents) and the planet by 2030 [4, 33]. Inherently, the JLA PSPs included here address 2 of the 17 SDGs, including #3 (‘good health and well‐being’) and #17 (‘partnership for the goals’) [4, 33]. We recognise limitations related to geographical bias present in our sample, as the majority of studies originate from the United Kingdom and Canada (*n* = 28, 67%), although not surprising given that the JLA is a UK‐based initiative [34]. The disproportionate representation of high‐income countries within this sample may have influenced common themes amongst priorities, which may have implications when assessing this study through the lens of global health equity. As such, several PSPs focus on specific conditions, such as asthma, diabetes and mental health disorders, that reflect greater global burden of disease‐related disability for children and adolescents amongst higher‐income countries [1, 2]; however, many risk factors and diseases contributing to high mortality and disability globally amongst children are not represented (e.g., lower respiratory infection, diarrhoea and malnutrition) [1, 3]. Furthermore, political, systemic and environmental factors that critically contribute to child health and well‐being as identified in the SDGs are minimally addressed (e.g., education, climate, poverty, hunger, inequalities, economy, clean water and sanitation) [4, 33]. The original conceptualisation of JLA PSPs, established in 2004, relied on the term ‘treatment uncertainties’ to define unanswered questions that are prioritised [11]. Although JLA now uses ‘evidence uncertainties’, the original term likely had historically biased priorities towards clinical treatments and interventions as opposed to environmental, socio‐economic and systems factors that critically influence child health, such as those identified in the SDGs. More broadly, included articles poorly reported race or ethnicity (12%–34%) and sex or gender (range from 26% to 57%). When reported, studies predominantly included individuals identifying as white and as female. Greater diversity and intersectionality are needed in future JLA PSPs—as is called for across child health research [35]—to ensure they uphold the JLA PSP core principle of inclusivity and begin to remedy demonstrated inequities. Participatory research methods, including but not limited to JLA PSPs, undertaken in partnership with equity‐deserving groups and children and youth, are a key approach to achieving true health equity [14, 35, 36, 37].

Our decision to focus on JLA PSPs aligns with the United Nations and UNICEF recommendations to empower young people in robust and inclusive decision‐making to accelerate transformative action towards the SDGs and to take measures to strengthen science‐policy‐society partnerships [4, 33]. JLA PSPs are considered deliberative, involving a higher degree of patient and public involvement than consultative approaches to priority setting [14]. Many priority‐setting efforts include only one participant group (e.g., researchers, health professionals and decision‐makers) and reveal different priorities depending on who is involved [9, 14]. For example, a review of participatory methods in mental health research priority setting revealed different priorities when the public and health professionals were involved (i.e., therapy, standards, education and psychology) versus only scientists (i.e., diagnosis, methods and standards of care) [9]. Partnership with people with lived experience throughout the research process (i.e., in priority setting, governance, conduct and mobilisation) can increase research relevance, efficiency and impact [38]. A 2021 scoping review identified that JLA PSPs represented the third most common method conducted or supported by research funding organisations to identify health research gaps, needs or priorities (28%), behind workshops or meetings (37%) and quantitative methods (32%), noting an overall trend towards greater engagement of people with lived experience [39]. Here, our decision to focus exclusively on child health JLA PSPs potentially limited the generalisability of our findings; however, that is offset by the strength of its equitable, patient‐oriented approach.

This review offers critical guidance to child health research funders and researchers. From our findings, it is clear that diverse multidisciplinary research across the translational research continuum—from basic science to health systems implementation and public health—is needed to adequately address all identified common priority themes. By focusing broadly on child health, our review took a disease‐agnostic versus disease‐specific approach. A key advantage is a broader perspective, illuminating opportunities for health research funders and/or decision‐makers to respond at a complex health systems level with the potential to benefit more children and families in a more cost‐effective manner [40]. This is needed to address identified top priority themes of ‘public health’ and ‘care coordination and health systems navigation’ and offers particular value given the common co‐occurrence of child health concerns or diagnoses, notably highlighted by ‘comorbidities’ as a priority theme. We acknowledge that this systems approach does not negate the need for disease‐specific research, particularly to investigate aetiology and risk factors, as well as biomedical interventions.

JLA PSPs are intended to highlight important areas for further research; however, they can potentially influence health research funding decisions through directed funding to identified priority areas (e.g., calls in themed areas, integrating priority topics into open calls) [15]. The priority theme areas identified in this review could also be integrated into organisational research strategies, using the priorities criterion for ranking the importance of research proposals, and/or targeted calls for shared priorities across areas of child health (e.g., systems‐level challenges). The potential influence of PSPs on health research funders appears to depend on alignment between a patient‐oriented priority and the remit, culture and values of the funding organisation [15]. This greatest influence may occur when PSPs are initiated and/or funded by charitable or not‐for‐profit organisations [15], as was the case for 40% of PSPs included here.

Interestingly, large health research funders in countries where most of the included PSPs were conducted (i.e., the United Kingdom, Canada and the United States) do not appear to report the proportion of funded research that addresses patient‐identified priorities. For example, the Canadian Institutes of Health Research (CIHR) reports funding investments by government priorities, institute and corporate‐led initiatives, and/or investigator‐initiated research [41]. CIHR's Strategy for Patient‐Oriented research (SPOR) program falls under its government priority funding investment area and emphasises the engagement of patients, caregivers and families as partners in the research process [42]. In the United States, the Patient‐Centered Outcomes Research Institute (PCORI) program is widely acknowledged for its promotion of patient‐centred comparative clinical effectiveness research [43]; however, PCORI's annual budget ($275.5–399 million USD) is minuscule (~1.3%) relative to research funding via the National Institutes of Health ($30.1 billion USD) [44, 45]. In the United Kingdom, a 2024 report from the UK Clinical Research Collaboration reported an annual £4 billion in clinical health research funding across 173 funding organisations. In this report, mention of patient and public involvement in research and/or patient priorities was variable across descriptions of health funding organisations [46]. The UK National Institute for Health and Care Research recently posted a specific funding call for research addressing any of the JLA PSPs (https://www.nihr.ac.uk/researchers/funding-opportunities/jla-psp-rolling-call.htm). Across all three countries, the greatest proportion of health research funding appears to be allotted to biomedical research [44, 45, 46, 47]. The priority themes identified in this review critically emphasise the need to grow the proportion of funding for other types of health research (e.g., clinical, health services and population health) to adequately reflect and address the priorities identified by children, adolescents, their families and health professionals.

While JLA PSPs are intended to identify research priorities, they can have a larger impact. Additional benefits of JLA PSPs include enhanced status and greater confidence for individuals involved, as well as strengthened relationships, partnerships, networks, directions for advocacy and connections with individuals and organisations [15]. Specifically, JLA PSP priorities herein identify areas of unmet or underserved need for children and their families [39]. These can be readily actioned by health professionals, the health system, policymakers and patient community groups and organisations in the absence of new research. This could include, for example, directly informing new educational activities or resources, new health service or program development and delivery, policy change and/or advocacy efforts in identified high priority areas. Effort is needed to ensure that PSPs are widely shared so that they can be actioned, which appears most successful when conducted in partnership with a relevant charity [15]. For example, one JLA PSP in paediatric chronic pain was effectively mobilised for widespread impact with patients and families, health professionals and policymakers [48]. This aligns with the cross‐cutting identified priority theme of ‘integrated knowledge translation’ and broader national and international efforts to incentivise and recognise the importance of research impact in spheres of clinical practice and policy [49, 50].

This review provides insight into common priority themes to improve child and adolescent health and well‐being. Transformative widespread improvement in child health requires addressing needs and gaps identified by people with lived experience. This necessitates asking and acting on the priorities of children, their families and the health professionals that support them.

## Author Contributions

**Jenna S. Jessa:** data curation, formal analyses, investigation, writing – original draft. **Muning (Linda) Zhang:** conceptualisation, data curation, formal analyses, investigation, methodology, writing – original draft. **Justin Bonhomme:** data curation, formal analyses, investigation, writing – original draft. **Dawn P. Richards:** conceptualisation, formal analyses, methodology, writing – review and editing. **Diane L. Lorenzetti:** conceptualisation, data curation, methodology, writing – review and editing. **Christine T. Chambers:** conceptualisation, writing – review and editing. **Kathryn A. Birnie:** conceptualisation, project administration, formal analyses, methodology, supervision, writing – original draft, writing – review and editing.

## Conflicts of Interest

The authors declare no conflicts of interest.

## Supporting information


**Supplemental Figure S1**: Cumulative number of JLA PSP child health articles per year.

**Supplemental 1:** Search Strategy.

**Supplemental Table S1:** Thematic synthesis of uncertainties.

**Supplemental Table S2:** JLA Priority Setting Partnership Study Characteristics.

## Data Availability

The data that support the findings of this study are available from the corresponding author upon reasonable request.
