# Supplementary figures and images for: Identifying Common Patient‐Oriented Priorities for Child and Adolescent Health Research and Care: A Systematic Review of Priority Setting Partnerships
Source: Health Expect. 2025 Jul 30;28(4):e70349. doi: 10.1111/hex.70349 (PMC12309730; doi:10.1111/hex.70349)

**
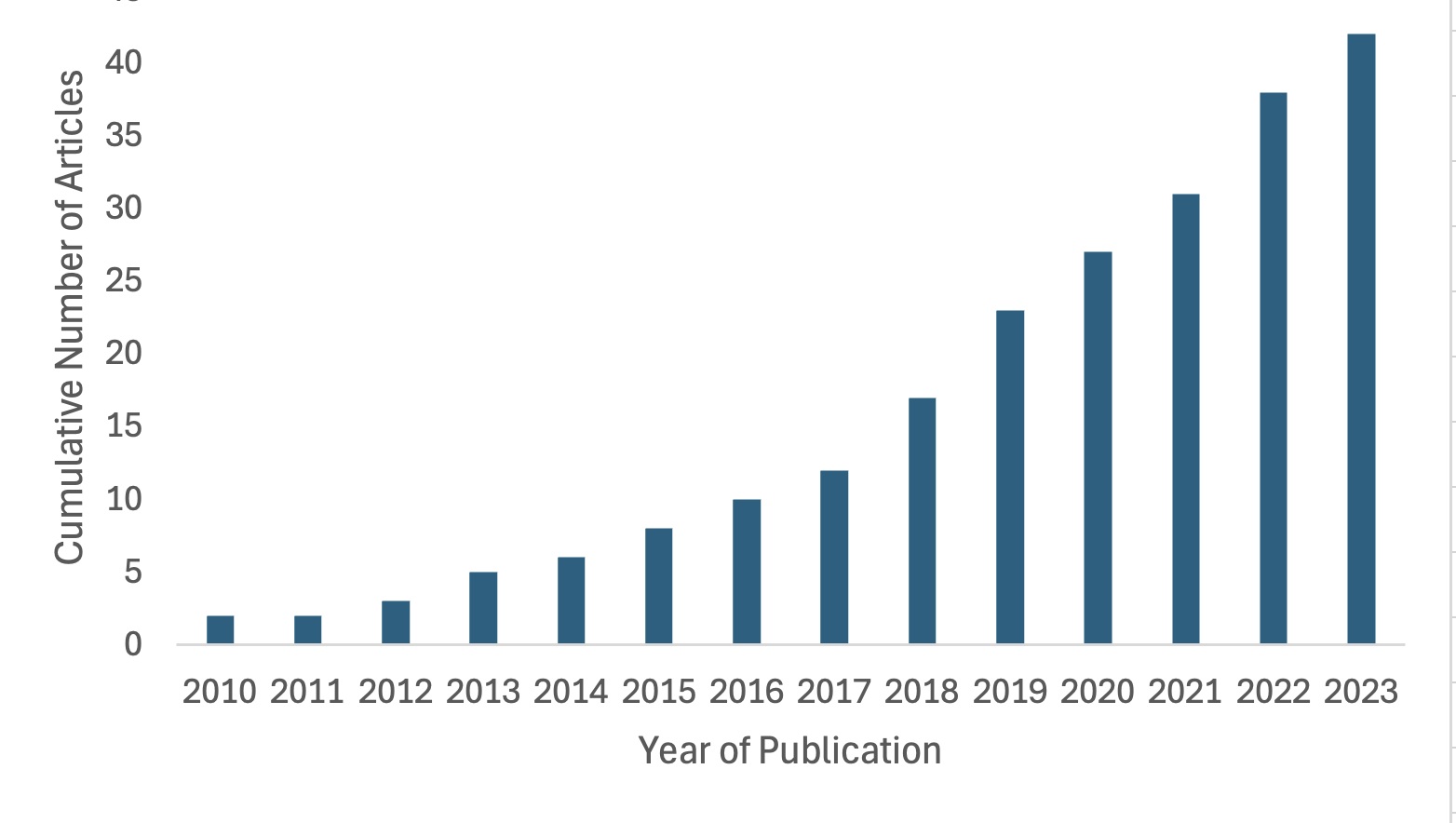
Supplemental Figure S1**. Cumulative number of JLA PSP child health articles per year.

Supplement: Supplementary file 1 — Supplemental Figure S1: Cumulative number of JLA PSP child health articles per year. [file HEX-28-e70349-s002.docx]
