# Supplementary material for: Identifying Common Patient‐Oriented Priorities for Child and Adolescent Health Research and Care: A Systematic Review of Priority Setting Partnerships
Source: Health Expect. 2025 Jul 30;28(4):e70349. doi: 10.1111/hex.70349 (PMC12309730; doi:10.1111/hex.70349)
Supplement: Supplementary file 2 — Supplemental 1: Search Strategy. [file HEX-28-e70349-s004.docx]

Supplemental 1. Search Strategy

1 James Lind Alliance.mp.

2 Priority Setting Partnership.mp.

3 1 or 2

4 priority setting.mp.

5 3 or 4

6 exp Child/ or "Congenital, Hereditary, and Neonatal Diseases and Abnormalities"/ or adolescent/ or exp pediatrics/ or child, abandoned/ or exp child, exceptional/ or child, orphaned/ or child, unwanted/ or minors/ or (pediatric* or paediatric* or child* or congenital* or preschool* or pre-school* or kindergarten* or kindergarden* or elementary school* or nursery school* or (day care* not adult*) or schoolchild* or toddler* or boy or boys or girl* or middle school* or pubescen* or juvenile* or teen* or youth* or high school* or adolesc* or pre-pubesc* or prepubesc*).mp. or (child* or adolesc* or pediat* or paediat*).jn.

7 5 and 6
