# Supplementary material for: Identifying Common Patient‐Oriented Priorities for Child and Adolescent Health Research and Care: A Systematic Review of Priority Setting Partnerships
Source: Health Expect. 2025 Jul 30;28(4):e70349. doi: 10.1111/hex.70349 (PMC12309730; doi:10.1111/hex.70349)
Supplement: Supplementary file 3 — Supplemental Table S1: Thematic synthesis of uncertainties. [file HEX-28-e70349-s001.docx]

| **Category** | **Priority** |
| --- | --- |
| General Symptom Treatment and Management    General Symptom Treatment and Management    General Symptom Treatment and Management    General Symptom Treatment and Management  General Symptom Treatment and Management  General Symptom Treatment and Management | 1. What are the effective ways of simplifying the treatment burden of people with Cystic Fibrosis? |
|  | 2. How can we relieve gastro-intestinal (GI) symptoms, such as stomach pain, bloating and nausea in people with CF? |
|  | 3. What is the best treatment for non-tuberculous mycobacteria (NTM) in people with CF (including when to start and what medication)? |
|  | 4 How should sickness and emergency situations be managed for patients with liver GSD? |
|  | 10 What are the risks and benefits of different options for overnight treatment for patients with liver GSD and how can we maximize safety? |
|  | 11 How to prevent and/or treat muscle problems in patients with liver GSD? |
|  | What is the best way to treat vascular or antibody-mediated acute rejection? |
|  | How can we prevent sensitisation in patients with a failing transplant, to improve their chances of another successful transplant (e.g. removal of the transplant, withdrawal of immunosuppressive medicines or continuation of these medicines?) |
|  | How can we improve transplant rates in highly sensitised patients? |
|  | 1 What is the short- and long-term efficacy of treatment methods for anorexia nervosa at different ages and which ones are the best? |
|  | 2 What are the most helpful and least helpful treatment elements as identified by recovered individuals, and what long-term outcomes do they perceive them to help with? |
|  | 4 Once recovered/discharged from services, what is the most effective way of preventing relapse for anorexia nervosa patients? |
|  | 8 What are the effects of repeated inpatient admissions for anorexia nervosa patients, and should treatment be different after repeated admissions? |
|  | 4. What is the best way to treat the symptoms of psoriasis: itching, burning, redness. scaling and "aking? |
|  | 7. Why do psoriasis treatments stop working well against psoriasis and when they stop working well. what’s the best way to regain control of the disease? |
|  | 5. How can outcomes in frail and/or elderly patients be improved after receiving anesthesia for surgery? |
|  | 9. How can anesthesiologists improve pain control after surgery? |
|  | 4 How can pain be most effectively managed in people with IBD? |
|  | 8 What is the association between IBD and fatigue and how should it be managed? |
|  | 1. What management strategy should be adopted for the treatment of acne in order to optimise short and long-term outcomes? |
|  | 3. What is the best treatment for acne scars? |
|  | 7. What is the best way of managing acne in mature women who may/may not have underlying hormonal abnormalities? |
|  | 10. How long do acne treatments take to work and which ones are fastest acting? |
|  | 1 What is the most effective treatment approach for hyperacusis in children? |
|  | 5 Which treatment approaches are most effective for different types or severities of hyperacusis? |
|  | 8 What management approach for hyperacusis is most effective for adults/children with autism? |
|  | 3 What are the key components of successful ‘self-management’ for a person with asthma? (British Thoracic Society) |
|  | 5 What is the most effective way of managing asthma triggers? (Asthma UK) |
|  | 1. Which wound care method obtains better outcomes (improved healing, decrease pain, improve quality of life, decrease smell, prevent infection) in patients with EB? Interventions include types of dressings (polyethylene, polyester plus petrolatum, hydrocolloid, collagen, hydrofiber, hydrogel, silicone…), topical antibacterial treatment (clorhexidine, bleach bath, vinegar bath, honey, antibiotics, silver dressings) and frequency of cure (daily or alternate days)? |
|  | 3. What is the best pain control strategy (analgesics, sedative drugs, addition of NaCl into the water) to decrease pain during wound care and bath in DEB patients? |
|  | 8. What interventions are effective for managing acute pain flares in children and adolescents with chronic pain? |
|  | 10.When are treatments for chronic pain in children and adolescents most effective (for example, after medical investigation is complete, or variation by type of treatment modality, or readiness of child/adolescent or family to engage in treatment)? |
|  | 10. What targeted treatments are effective and have fewer short-term and long-term side-effects? |
|  | 9) Determine the best treatments for pain due to dystonia in CP |
|  | 3) What are best practices and support strategies for Indigenous parents, families, and children and youths on the GPIU? |
|  | 8) What are effective strategies to mitigate the impacts of prolonged inpatient hospitalizations on GPIUs? (eg, addressing unmet needs, prolonged separation from family) |
|  | 1. Safer anesthesia for children (e.g., avoiding breathing problems) |
|  | 3. Improving perioperative care for children with chronic conditions undergoing surgery (e.g., heart, lung, epilepsy, diabetes, obesity) |
|  | 5 Improving children's recovery and pain management at home throughout the surgical experience |
|  | 9 Improving pain management in children with chronic pain |
|  | 4. What is the best way to treat the symptoms of psoriasis: itching, burning, redness, scaling and flaking? |
|  | 20. What triggers a sudden flare-up of psoriasis and how long do these usually last? |
|  | 1. What are the optimal pelvic floor muscle training protocols (frequency and duration of therapy) for the treatment of different patterns of urinary incontinence? |
|  | 3. What is best practice for the treatment of combined stress urinary incontinence and detrusor over activity? |
|  | 5. Which treatment is most effective for the reduction of urinary frequency and urgency? |
|  | 7. What is best practice for the management of stress urinary incontinence following failed tension free vaginal tape surgery? |
|  | 8. What are the most effective treatments of daytime urinary incontinence in children? |
|  | 3. What is the best treatment plan for uveitis in JIA, and are there factors that predict its effectiveness? |
|  | 4. Why are children with JIA fatigued more quickly, what can be done about it, and how can one best cope with the fatigue in daily life? |
|  | 3. What is the short-term and long-term clinical and cost effectiveness of orthopaedic lower limb surgery for children with Cerebral Palsy who can walk (considering best timing and technique)? |
|  | 10. What is the best management for hip displacement in children with Cerebral Palsy? |
|  | Is it necessary to continue treatment for patients with lichen sclerosus who do not have any symptoms and/or signs of disease activity? |
|  | Are there ways of restoring sight loss for people with AMD? |
|  | What are the best enablement strategies for people with AMD? |
|  | How can cerebral visual impairment be identified, prevented and treated in children? |
|  | What is the most effective management for dry eye and can new strategies be developed? |
|  | What is the most effective management of ocular complications associated with Stevens Johnson Syndrome? |
|  | Can severe ocular surface disease in children, such as blepharokeratoconjunctivitis and vernal keratoconjunctivitis be managed better? |
|  | What are the most effective treatments for glaucoma and how can treatment be improved? |
|  | How can loss of vision be restored for people with glaucoma? |
|  | What is the most effective way to support patients with inherited retinal disease? |
|  | Would having a treatment for an inherited retinal disease preclude a patient from having another treatment? |
|  | What are the most effective treatments and rehabilitation for optic neuropathies, eg, Leber’s hereditary optic neuropathy and anterior ischaemic optic neuropathy? |
|  | What rehabilitation or treatment methods are most effective for vision loss following brain damage due to stroke, brain injury, cerebral vision impairment, tumours and dementias? |
|  | What can be done to help ocular cancer sufferers? |
|  | How can follow-up for ocular complications be managed in patients with ocular melanoma? |
|  | What is the best management of metastatic choroidal melanoma? |
|  | What is the most effective treatment for primary ocular melanoma? |
|  | What are the most effective treatments for ocular and orbital inflammatory diseases? |
|  | What is the most effective treatment for exotropia and when should it be delivered? |
|  | 2. What are the most effective interventions to support sleep in children and young people with neurological conditions? |
|  | 5. What are the most effective medicinal and non-medicinal treatments to manage distressing symptoms (e.g. pain, irritability) in children and young people suffering life limiting neurological conditions? |
|  | 9 How can the longevity of the Fontan circulation be prolonged and the impact of complications (eg, liver, protein-losing enteropathy, renal, endocrine, fertility) be reduced? |
|  | 7) What are effective interventions to help children manage their emotions? |
| Non-pharmacological interventions  Non-pharmacological interventions  Non-pharmacological interventions  Non-pharmacological interventions  Non-pharmacological interventions  Non-pharmacological interventions | 6. What effective ways of motivation, support, and technologies help people with CF improve and sustain adherence to treatment? |
|  | 7. Can exercise replace chest physiotherapy for people with CF? |
|  | 5 What is the best way to start dietary treatment, finding the optimal doses, and to administer the diet for patients with liver GSD? |
|  | 8 How to manage diet regimen in relation to "before, during and after" physical exercise (sport, playing) for patients with liver GSD? |
|  | 9 What are the long-term complications (liver, renal, gut) of a diet rich in uncooked cornstarch and/or high protein and should the diet be adjusted to prevent complications in liver GSD? |
|  | 1. Does the timing and intensity of therapies (eg, physical, occupational and speech and language therapy, ‘early intervention’, providing information, etc) alter the effectiveness of therapies for infants and young children with neurodisability, including those without specific diagnosis? What is the appropriate age of onset/strategies/dosage/direction of therapy interventions? |
|  | 2. To improve communication for children and young people with neurodisability: (A) what is the best way to select the most appropriate communication strategies? And (B) how to encourage staff/carers to use these strategies to enable communication? |
|  | 3. Are child-centred strategies to improve children’s (ie, peers) attitudes towards disability (eg, buddy or circle of friends, etc) effective to improve inclusion and participation within educational, social and community settings? |
|  | 4. Does appropriate provision of wheelchairs to enable independent mobility for very young children improve their self-efficacy? |
|  | 5. Are counselling/psychological strategies (eg, talking therapies) effective to promote the mental health of children and young people with neurodisability? |
|  | 8. What strategies are effective to improve engagement in physical activity (to improve fitness, reduce obesity, etc) for children and young people with neurodisability? |
|  | l. Do lifestyle factors such as diet. dietary supplements, alcohol, smoking, weight loss and exercise play a part in treating psoriasis? |
|  | 5. How well do psychological and educational interventions work for adults and children with psoriasis? |
|  | 3 What role does diet have in the management of mildly active or inactive ulcerative colitis or Crohn’s disease to achieve normal daily activities and symptom control? |
|  | 7 What is the optimal dietary therapy (liquid enteral diet and/or reintroduction diet) and duration to achieve mucosal healing in active IBD and/or remission either as a primary or adjunctive treatment? Is there a difference between adults and children? |
|  | 2 What interventions increase physical activity in children? |
|  | 5 What interventions promote social skill development? |
|  | 8 What are effective strategies for behaviour management in children? |
|  | 9 What nutritional factors affect child behaviour? |
|  | 3. What are the effects of multimodal therapy? |
|  | 6. What are the effects of parental support programmes? |
|  | 7. What are the effects of supported conversation? |
|  | 8. What are the effects of computer-aided working memory training? |
|  | 9. What are the effects of psychoeducative treatment? |
|  | 3. What role does diet have in the management of pediatric IBD? |
|  | 7 Which psychological therapy (eg, counselling, cognitive–behavioural therapy, mindfulness) is most effective for hyperacusis? |
|  | 9 What is the best way of using sound in therapy for hyperacusis? |
|  | 10 Which self-help interventions are effective for hyperacusis? |
|  | 6 What is the role of complementary therapies in asthma management? (Asthma UK) |
|  | 7 What are the benefits of breathing exercises as a form of physical therapy for asthma? (Asthma UK) |
|  | 10 What are the most effective psychological interventions for adults with asthma? (British Thoracic Society |
|  | 7. Which is the most effective method in avoiding or delaying syndactyly in patients with DEB? Including different types of bandages, dressings, gloves and splints, physiotherapy and occupational therapy. |
|  | 3. What physical and psychological treatments are effective for improving pain and function in children and adolescents with chronic pain (for example, functional outcomes including quality of life, depression, fatigue, sleep, acceptance, concentration, resilience, coping, self-management)? |
|  | 5. What is the best way to practice your favorite sport safely? |
|  | 2. What interventions, including self-care, can reduce or reverse adverse short-term and long-term effects of cancer treatment? |
|  | 6. What are the most effective strategies to ensure that young people who are treated outside of a young person’s principal treatment centre receive appropriate practical and emotional support? |
|  | 2) Assess rehabilitation, psychological, and environmental approaches to manage dystonia |
|  | 5) What are effective support strategies for parents, families, and children and youths hospitalized on the GPIU? (eg, support groups, private rooms/sleeping arrangements, breastfeeding support, physical activity, making the ward more adolescent-friendly, screen time) |
|  | 1. Are oromotor therapy techniques effective and cost- effective in improving eating and drinking and health outcomes for children and young people with non-progressive neurological conditions? |
|  | 6. Is graded exposure effective and cost-effective in improving health and well-being outcomes for children with behavioural and/or sensory feeding difficulties? |
|  | 1. Do lifestyle factors such as diet, dietary supplements, alcohol, smoking, weight loss and exercise play a part in treating psoriasis? |
|  | 5. How well do psychological and educational interventions work for adults and children with psoriasis? |
|  | 7. What is the influence of nutrition on JIA, and can a diet help? |
|  | 10. What is the influence of sports and exercise on JIA and vice versa? |
|  | 2. What should children’s rehabilitation following orthopaedic surgery to the lower limbs include, how long is it expected to last and how does it affect the result of treatment? |
|  | 5. What is the role of pre-operative rehabilitation in children presenting with lower limb orthopaedic conditions? |
|  | 7. Can surveillance and non-surgical treatment (physiotherapy, botulinum toxin injections, functional electrical stimulation, orthotics, casting) prevent the development of deformity requiring surgery in children with Cerebral Palsy? |
|  | What dietary supplements should my child be taking? |
|  | Does exercise enhance the immune system of children with Down syndrome? |
|  | What complementary therapies are beneficial for my child? |
|  | What complementary therapies are beneficial for my child? |
|  | What dietary supplements should my child be taking? |
|  | What complementary therapies are beneficial for my child? |
|  | 2 (12)Can a non-invasive therapy be developed for wet AMD? |
|  | Can dietary factors, nutritional supplements, complementary therapies or lifestyle changes prevent or slow the progression of AMD? |
|  | Can non-surgical therapy be developed for Fuchs’ corneal dystrophy? |
|  | Can diet or lifestyle changes prevent uveitis from developing? |
|  | 3. Does a high protein feed formula combined with early mobilization reduce muscle wasting in children on breathing machines? |
|  | 7. Does giving probiotics (healthy bacteria) reduce the risk of hospital acquired infections in children on breathing machines? |
|  | 9. Which psychological interventions are most effective in children and young people who have functional neurological disorders? |
|  | 10. What are the best non-medicinal interventions (including therapies, orthotics e.g. splints, high and low technology supports) for children and young people with motor disorders? |
|  | 3. How can we optimise a healthy diet? |
|  | 8 How can less invasive interventions be performed for CHD with the same outcomes as open-heart surgery? |
|  | 4) What are the effects of food intake on mood in children? |
|  | 7. Does a structured training programme, medicines and/or surgery speed up the achievement of continence (either/or faecal or urinary) for children and young people with neurodisability? |
|  | 10. What is the long-term safety, effectiveness and sustainability of behavioural strategies and/or drugs (eg, melatonin) to manage sleep disturbance in children and young people with neurodisability (outcomes include time to onset, duration, and reducing impact on family)? |
|  | 3. What is the most effective way to manage the transition from tube feeding to oral feeding in terms of health and well- being outcomes in premature infants? |
|  | What rehabilitation programmes are best for the management of distorted vision from retinal diseases? |
|  | 5. What are the most effective medicinal and non-medicinal treatments to manage distressing symptoms (e.g. pain, irritability) in children and young people suffering life limiting neurological conditions? |
| Biomedical Interventions | How can surgical techniques be improved to save sight for eyes damaged by injury? |
|  | 8. Which antibiotic combinations and dosing plans should be used for CF exacerbations and should antibiotic combinations be rotated? |
|  | 9. Is there a way of reducing the negative effects of antibiotics, for example resistance risk and adverse symptoms in people with CF? |
|  | 3. What is the best treatment for non-tuberculous mycobacteria (NTM) in people with CF (including when to start and what medication)? |
|  | 3 How should optimal metabolic control both clinically and biochemically (like lactate, ketones, and/or lipids) be achieved in liver GSD? |
|  | How can immunosuppression be personalised to the individual patients to improve the results of transplantation? |
|  | How can we encourage tolerance to the transplant to prevent or reduce the need for immunosuppression? (e.g. by use of T-regulatory cells, induction of haemoxygenase 1) |
|  | What is the best combination of immunosuppressive drugs following kidney transplantation? (e.g. azathioprine or mycophenolate, belatacept, generic or proprietary (brand-name) drugs) |
|  | What techniques to preserve, condition and transport the kidney before transplantation allow increased preservation times and/or improve results? (e.g. machine perfusion, normothermic reconditioning, addition of agents to the perfusate) |
|  | 6. What is the (long-term) comparative safety and effectiveness of medical and surgical spasticity management techniques (botulinum neurotoxin A, selective dorsal rhizotomy, intrathecal baclofen, orally administered medicines) in children and young people with neurodisability? |
| Biomedical Interventions | 7. Does a structured training programme, medicines and/or surgery speed up the achievement of continence (either/or faecal or urinary) for children and young people with neurodisability? |
|  | 10. What is the long-term safety, effectiveness and sustainability of behavioural strategies and/or drugs (eg, melatonin) to manage sleep disturbance in children and young people with neurodisability (outcomes include time to onset, duration, and reducing impact on family)? |
|  | 6. What is the impact of reducing opioids (a type of medication that reduces pain, like morphine) during anesthesia on patient outcomes and opioid dependence after surgery? |
|  | 6 What is the best treatment for controlling diarrhoea and/or incontinence symptoms in people with IBD, including novel pharmacological and nonpharmacological options? Is high-dose loperamide safe and effective in the treatment of diarrhoea in IBD? |
|  | 2. What is the correct way to use antibiotics in acne to achieve the best outcomes with least risk? |
|  | 5. What is the correct way to use oral isotretinoin (Roaccutane) in acne in order to achieve the best outcomes with least risk of potentially serious adverse effects? |
|  | 8. What is the best topical product for treating acne? |
|  | 9. Which physical therapies, including lasers and other light based treatments, are safe and effective in treating acne? |
|  | 1. Is there a risk that medication with methylphenidate during childhood will lead to the development of drug dependence later in life? |
|  | 4. Which of the two pharmaceuticals, atomoxetine or methylphenidate, is most effective, with fewer side effects? 5. What are the effects of methylphenidate medication in substance abusers? |
|  | 10. What are the effects of treatment of sleep disorders with melatonin? |
| Biomedical Interventions | 1 (a) What are the adverse effects associated with long-term use of short- and long-acting bronchodilators; inhaled and oral steroids; and combination and additive therapies in adults and children aged 12 years old and over? |
|  | 1 (b) What are the adverse effects associated with long-term use of short- and long-acting bronchodilators; inhaled and oral steroids; and combination and additive therapies in children? (Asthma UK) |
|  | 2. What is the best treatment to control itch in DEB patients (sedating antihistaminics, non-sedating antihistaminics, topical menthol, topical corticosteroids, moisturizers, doxepin, gabapentine, cyclosporine, dronabinol, ondansetron)? |
|  | 3. How can pain best be recognized and be treated (with medication), and what action can a patient take him/herself? |
|  | 4. How can pills be manufactured in such away that they are easy to take? (i.e.shape, color, taste) |
|  | 5. Is cutting tongue tie effective and cost-effective in terms of feeding outcomes in infants with tongue tie? |
|  | 7. Is feeding via a gastrostomy tube effective in improving health and well-being outcomes of (a) children with neurological conditions and dysphagia and (b) parents of children with neurodisability and dysphagia? |
|  | 10 More child friendly medications, for example, better tasting, easier to take |
|  | 11. Are oral and biological treatments for psoriasis safe to use for people with psoriasis or their partner if they are trying to have a baby, and are they safe to use during pregnancy? |
|  | 12. What are the long-term benefits and risks of oral and biological psoriasis treatments? |
|  | 14. Can oral and biological treatments for psoriasis be combined safely and does combining them work well? |
|  | 19. What is the best and most cost-effective way of monitoring an oral or biological therapy for psoriasis, e.g. clinical review, blood tests and measurement of medicine/antibodies levels? |
| Biomedical Interventions | 4. What catheter regimens are most effective in preventing urinary tract infections in patients using intermittent self-catheterization for the management of a neurogenic bladder?/What is the effectiveness and safety of prophylactic versus symptomatic antibiotic therapy in patients with neurogenic bladder dysfunction using intermittent self-catheterization |
|  | 9. Are disposable catheters more or less acceptable than reusable catheters in terms of effective bladder management, patient experience, and urinary tract infections? |
|  | 10. In women with prolapse and stress urinary incontinence, should suburethral tapes be inserted at the same time as repairing the prolapse? |
|  | 8. What are the short and long term side effects/consequences of the drugs taken for JIA? |
|  | 6. What is the short-term and long-term clinical and cost effectiveness of Selective Dorsal Rhizotomy (SDR) in children with Cerebral Palsy who can walk? |
|  | If my child is diagnosed with a heart condition in the womb, are there any medications I can take to help my baby? |
|  | Is it okay for my child to get vaccinated? |
|  | What type of operations are available for babies in the womb to reduce the effect of their condition? |
|  | What is the best age for children with a cleft to have surgery? |
|  | What surgical treatments should be offered for lichen sclerosus? |
|  | How can cataract surgery outcomes be improved? |
|  | How safe and effective is laser assisted cataract surgery? |
|  | Can retinal detachment be prevented after cataract surgery? |
|  | What are the outcomes for cataract surgery among people with different levels of cognitive impairment (all causes excluding dementia, stroke, neurological conditions, head injuries)? |
| Biomedical Interventions | How can the rejection of corneal transplants be prevented? |
|  | Can the outcomes of corneal transplantation be improved? |
|  | How can immunotherapy be used to fight metastatic ocular melanoma? |
|  | What is the efficacy and safety of anti-VEGF agents in the treatment of retinopathy of prematurity? |
|  | 5. When should intravenous nutrition start in very underweight critically ill children who DO NOT have a working digestive system? |
|  | 6. What are the safest and most effective anti-seizure medications for seizures in new-born babies less than 28 days old? |
|  | 7. Which medications should be used, and in what sequence, in the management of muscle stiffness (hypertonia) in children and young people? |
|  | 8. Are medications (e.g. antibiotics and/or immune treatments) effective in the management of PANS/PANDAS? (Paediatric Acute-onset Neuropsychiatric Syndrome/Paediatric Acute-onset Neuropsychiatric Disorders Associated with Streptococcal infection) |
|  | 19. What is the impact of vaccinations on children’s health (upto date, barriers, positive messages)? |
|  | 1 How can damage to organs (eg, heart, brain, lung, kidney, bowel) during heart surgery in children with CHD be minimised to reduce complications, especially in those who require multiple operations? |
|  | 1 What are the best options (eg, gene therapy or enzyme replacement therapy) for achieving sufficient amount of working enzyme in patients with liver GSD? |
|  | 3) Compare effectiveness of pharmacologic and surgical treatments for dystonia (including evaluation of side effects, a person’s overall function, and effect on individualized goals) |
|  | Are there effective topical treatments other than topical steroids in the treatment of lichen sclerosus? |
| Biomedical Interventions | 5. What are the most effective medicinal and non-medicinal treatments to manage distressing symptoms (e.g. pain, irritability) in children and young people suffering life limiting neurological conditions? |
| Public Health | 3 Of those diagnosed with anorexia nervosa, what are the rates of recovery, relapse, dropouts, and length of treatment across Canada? |
|  | 6 How could the system better support individuals with anorexia nervosa through transition periods so that care is streamlined and easier to navigate? |
|  | 10 What are the wait times across Canada for adolescent girls and women suffering from anorexia nervosa? Are they equitable? How can we decrease wait times for services? |
|  | 6 What is the impact of parental stress on children? |
|  | 10 How much screen time is appropriate for children? |
|  | 8 Miscellaneous How to improve collaboration across the different top centres caring for retinoblastoma: forming an international consortium, a unified registry and combined trials, instead of the current air of competition? |
|  | 10 Global health How can optimal retinoblastoma care be delivered in low-resource settings (including rural and remote communities)? |
|  | 2 What is the prevalence of hyperacusis in a general population and other specific populations (eg, people with autism, mental health issues, learning disabilities, hearing loss)? |
|  | 4. What strategies improve access and delivery of evidencebased treatments, and coordination of care, for all Canadian children and adolescents with chronic pain and their families, with a view to reduce disparities? |
|  | 6. What strategies effectively increase governmental and health care organizational financial support for evidence-based pediatric chronic pain care in Canada? |
|  | 3. What are the best strategies to improve access to clinical trials? |
| Public Health | 4) How can we ensure that health care delivery in the hospital meets the needs of children and youths with developmental disabilities on the GPIU? |
|  | 5. This theme included, how nurses can help children with developmental delays; support children to stay out of hospital; increase attendance through local community-based services; and nurse-led clinics |
|  | 6. Improvements in care for children in remote and regional Australia |
|  | 9. What is the influence of JIA on future opportunities regarding school results, work and relationships? |
|  | Is there an increased number of hospital admissions during winter with children with heart defects? |
|  | How many children have heart surgery and how many survive? |
|  | How many children have surgery and how many survive? |
|  | What is the rate of reoccurrence of cleft lip with or without cleft palate among siblings? |
|  | Why is glaucoma more aggressive in people of certain ethnic groups, such as those of West African origin? |
|  | What are the barriers that prevent diabetic patients having regular eye checks? |
|  | 6. What is the definition of feed intolerance in children on breathing machines? |
|  | 1. How can we ensure health services are appropriate for community needs and accessed by those that need them? |
|  | 2. What types of services (voluntary/cultural/youth) are needed to promote health and wellbeing? |
|  | 4. What are the barriers to a healthy lifestyle (individual, community, structural)? |
|  | 5. How does the quality of people’s housing afect their health? |
|  | 7. How can we reduce exposure to pollution? |
| Public Health | 8. How best can we improve sustainable travel and encourage active travel? |
|  | 9. How do we encourage children to be physically active? |
|  | 10. What are the barriers that stop children from being physical active? 11. What elements of a child’s home environment are most important for health and wellbeing? |
|  | 16. What is childhood and how does it afect health and wellbeing? |
|  | 17. How can we ensure access/encourage to high quality natural environments? |
|  | 20. What is important for health and health conditions? |
|  | 21. What are the barriers (individual, community, organisational environments) that stop people leading healthy lifestyles? |
|  | 22. What is important for a healthy mouth for children? |
|  | 24. What is the impact of screen time on children’s health? |
|  | 25. How we can encourage diferent ages and communities to work together? |
|  | 26. How does consumerism efect our health and wellbeing? |
|  | 27. How do we build inclusive environments for children regardless of culture, ethnicity, disability and background? |
|  | 3) What are the impacts of COVID-19-related closures/lockdowns (i.e., school, activities, playgrounds) on children's physical and mental health? |
|  | 5) How does screen time impact a child's physical, social, and behavioural development? |
|  | 9) What are the effects of social media on children? |
|  | 1) How can we raise awareness, increase community inclusion and reduce the exclusion and isolation of young people with chronic conditions anddisabilities in all aspects of life? |
| Public Health | 2 )How can young people with chronic conditions and disabilities access appropriate treatment and support quickly and easily? |
|  | 4) How can we support young people living with chronic conditions and/or disabilities during school, work and study? |
|  | 5) How can we improve the effectiveness of treatments and support for chronic conditions and disabilities? |
|  | 7) How can we make treatment more affordable and address the financial support needs of young people with chronic conditions and disabilities? |
|  | 9) How can we support the health and well‐being of parents, carers and families of young people living with chronic conditions and disabilities? |
| Screening, assessment, and monitoring | Can more effective diagnostic tools be developed for assessing the vitreous and eye floaters? |
|  | 7 What is the role for new methods for monitoring metabolic control (like noninvasive continuous glucose and lactate measurements, new biomarkers) for patients with liver GSD? |
|  | Can we improve monitoring of the level of immunosuppression to achieve better balance between risk of rejection and side effects? (e.g. T-cell or B-cell ELISPOT, point-of-care tacrolimus monitoring, MMF monitoring) |
|  | 7. What preparation, treatment, or assessment before receiving anesthesia for surgery improves patient outcomes? |
|  | 2 What are the optimal markers/combinations of markers (clinical, endoscopic, imaging, genetics, other biomarkers) for stratification of patients with regards to (a) disease course, (b) monitoring disease activity and (c) treatment response? |
|  | (8) What is the best way to assess learning difficulties in children and young people? |
|  | 7 What are effective methods for screening for developmental delay in children? |
|  | 5. How can we better define the role of, and improve access to, newer noninvasive, less costly, biomarkers of IBD endoscopic activity? |
| Screening, assessment, and monitoring | 6. How can we increase the knowledge and/or awareness around pediatric IBD so that diagnosis is not delayed? |
|  | 10. What is the optimal approach to diagnosis (education, psychological support, diagnostic tests) in pediatric patients with IBD? |
|  | 1 Diagnosis How to increase early diagnosis of retinoblastoma (i.e., decrease age or stage at diagnosis)? |
|  | 2 Second cancer What second cancer screening is optimal for heritable retinoblastoma survivors (including whole body magnetic resonance imaging)? |
|  | 4) Improve the clinical consistency of dystonia diagnosis and severity assessments |
|  | 2. Does cervical auscultation (listening to the sounds that accompany swallowing using a stethoscope placed on the neck) improve (a) identification of swallowing difficulties in children, and (b) carer’s understanding of children’s swallowing when they listen to the auditory feedback while their child is swallowing? |
|  | 9. What is effectiveness of the Neonatal Oral- Motor Assessment Scale in identifying and managing sucking difficulties in infants? |
|  | 1. What are the best ways to measure outcome following lower limb orthopaedic surgery in children? |
|  | 8. What is the best method of screening for Developmental Dysplasia of the Hip (DDH) in terms of clinical and cost effectiveness? |
|  | What is the best way to diagnose lichen sclerosus (diagnostic criteria)? |
|  | Which aspects of lichen sclerosus should be measured to assess response to treatment? |
|  | 6 What is the most effective way to detect and monitor the progression of early AMD? |
|  | What is the best measure of visual disability due to cataract? |
|  | How can cerebral visual impairment be identified, prevented and treated in children? |
| Screening, assessment, and monitoring | How do we improve screening and surveillance from the antenatal period through to childhood to ensure early diagnosis of impaired vision and eye conditions? |
|  | How can retinoblastoma be identified, prevented and treated in children? |
|  | What can be done to improve early diagnosis of sight-threatening glaucoma? |
|  | What is the most effective way of monitoring the progression of glaucoma? |
|  | How can glaucoma patients with a higher risk to progress rapidly be detected? |
|  | Is a genetic (molecular) diagnosis possible for all inherited retinal diseases? |
|  | Can the diagnosis of inherited retinal diseases be refined so that individuals can be given a clearer idea about their specific condition and how it is likely to progress? |
|  | With regard to inherited retinal diseases what is the role of prenatal and preimplantation diagnosis in helping parents make informed choices? |
|  | What is the most effective way to assess vision in patients with neurological visual impairment ie, stroke, dementia and cerebral/ cortical visual impairment? |
|  | Can the early stages of optic neuropathy be detected? |
|  | What are the most effective detection and screening methods for follow-up to detect metastasis of ocular melanoma? |
|  | Can the severity of ocular and orbital inflammatory disease in an individual be predicted? |
|  | Can early detection methods be developed for ocular and orbital inflammatory diseases? |
|  | Does early diagnosis of refractive error improve long-term prognosis and promote faster, more effective treatment? |
| Screening, assessment, and monitoring | How can the functional effects of surgical treatment for squint best be assessed? |
|  | Could the accurate testing of refractive error be made less dependent on a subjective response ie, the person’s own response? |
|  | Is there a way to improve screening of premature babies for retinopathy of prematurity? |
|  | Can a retinal vein occlusion be predicted and prevented? |
|  | 1. Can energy needs of babies on breathing machines be more accurately measured using indirect calorimetry |
|  | 2 How can prenatal and postnatal screening strategies (eg, scans, pulse oximetry, novel techniques) be improved to achieve greater accuracy, avoid late diagnosis and reduce complications from CHD? |
|  | 10) What are the early predictors of ADHD in children? |
|  | 3) How can we improve the process of diagnosing chronic conditions and disabilities? |
| Prevention and Early Intervention | What causes retinal detachment and can it be prevented? |
|  | How can epiretinal membrane/fibrosis be prevented or treated? |
|  | Are there methods to prevent and improve the treatment of macular holes? |
|  | 4. Which therapies are effective in delaying or preventing progression of lung disease in early life in people with CF? |
|  | 5. Is there a way of preventing CF related diabetes (CFRD) in people with CF? |
|  | 4 Once recovered/discharged from services, what is the most effective way of preventing relapse for anorexia nervosa patients? |
|  | 1. Does the timing and intensity of therapies (eg, physical, occupational and speech and language therapy, ‘early intervention’, providing information, etc) alter the effectiveness of therapies for infants and young children with neurodisability, including those without specific diagnosis? What is the appropriate age of onset/strategies/dosage/direction of therapy interventions? |
| Prevention and Early Intervention | 2. Does treating psoriasis early (or proactively) reduce the severity of the disease, make it more likely to go into remission, or stop other health conditions developing? |
|  | (4) Which early interventions are effective for children and young people with learning difficulties, at what ages and stages are they best introduced and what are the long-term outcomes? |
|  | (7) How can we best identify early features, symptoms and signs of learning difficulties among children, young people and their families/carers? |
|  | 4. What is the best way of preventing acne? |
|  | 4 What are effective interventions for obesity prevention in young children? |
|  | 2. Can IBD be prevented? |
|  | 5. How effective is a "tumor early diagnosis protocol" in patients with DEB to decrease mortality, amputations and disability? |
|  | 2. Does treating psoriasis early (or proactively) reduce the severity of the disease, make it more likely to go into remission or stop other health conditions developing? |
|  | 4 At what point in the care pathway (eg, crisis intervention, prevention, engagement, treatment, maintenance, and recovery) are digital interventions most safe and effective? |
|  | Would early intervention, eg, tummy time, creeping, and crawling, enhance my child’s development? |
|  | Can you pick up heart defects during pregnancy and reduce the damage? |
|  | What is the best way to prevent and manage anatomical changes caused by lichen sclerosus? |
|  | Can lichen sclerosus be prevented from occurring and what are the trigger factors? |
|  | 1 Can a treatment to stop dry AMD progressing and/or developing into the wet form be devised? |
| Prevention and Early Intervention | How can AMD be prevented? |
|  | How can cataracts be prevented from developing? |
|  | Can the return of cloudy or blurred vision after cataract surgery known as posterior capsule opacity (PCO) or secondary cataract be prevented? |
|  | How can cataract progression be slowed down? |
|  | How can cataract be prevented in children? |
|  | What are the causes of coloboma and microphthalmia/ anophthalmia and how can they be prevented? |
|  | How can retinoblastoma be identified, prevented and treated in children? |
|  | Can corneal infections be prevented in high-risk individuals such as contact lens wearers? |
|  | What is the cause of keratoconus and can it be prevented? |
|  | How can glaucoma be prevented? |
|  | Can a treatment to slow down progression or reverse sight loss in inherited retinal diseases be developed? |
|  | How can sight loss be prevented in an individual with inherited retinal disease? |
|  | How can optic neuropathies be prevented, for example anterior ischaemic optic neuropathy, Leber’s hereditary optic neuropathy, optic neuritis and other optic neuropathies? |
|  | What are the causes of ocular cancer and how can they be prevented? |
|  | Is it possible to prevent further occurrences of retinal damage caused by toxoplasmosis? |
|  | What medications best prevent the development of eye disease in Behcets? |
| Prevention and Early Intervention | How can the development of binocular vision in young children with squint and amblyopia be promoted, and would the same approach work in older individuals without inducing intractable diplopia? |
|  | How can myopia be prevented? |
|  | What are the best methods to prevent retinopathy of prematurity? |
|  | How can sight loss from diabetic retinal changes be prevented and reduced? |
|  | 1. Can early therapy interventions improve functional and developmental outcomes in babies experiencing brain injury during pregnancy or infancy? |
|  | 4 How can the frequency or need for reoperations be reduced for people with CHD (eg, improved valve/conduit longevity or that grow with the patient)? |
|  | What medications best prevent the development of eye disease in Behcets? |
|  | How can the development of binocular vision in young children with squint and amblyopia be promoted, and would the same approach work in older individuals without inducing intractable diplopia? |
|  | How can myopia be prevented? |
|  | What are the best methods to prevent retinopathy of prematurity? |
|  | How can sight loss from diabetic retinal changes be prevented and reduced? |
|  | 1. Can early therapy interventions improve functional and developmental outcomes in babies experiencing brain injury during pregnancy or infancy? |
| Prevention and Early Intervention | 4 How can the frequency or need for reoperations be reduced for people with CHD (eg, improved valve/conduit longevity or that grow with the patient)? |
| Prognosis and Disease Course | What are the long-term health risks to the living kidney donor? |
|  | 2 What are the most helpful and least helpful treatment elements as identified by recovered individuals, and what long-term outcomes do they perceive them to help with? |
|  | 10. What’s the best way to treat sudden "are ups of psoriasis? |
|  | 10. What are the common long-term side effects of anesthesia after surgery? |
|  | 9 Does early surgery or later surgery for terminal ileal Crohn’s disease result in better outcomes (quality of life, cost effectiveness)? |
|  | 4. What triggers flare ups in pediatric IBD? |
|  | 7. What are the long-term effects of medications used to treat pediatric IBD? |
|  | 8. How does an early diagnosis of pediatric IBD in childhood/teenagers impact the lifelong course (prognosis) of the disease? |
|  | 6 Psychosocial What is the effect of enucleation and vision loss on retinoblastoma survivors? |
|  | 6. What are the long-term results of syndactyly surgery? Which is the best technique? How often should it be performed? |
|  | 1. What treatments or strategies effectively prevent acute pain from becoming chronic in children and adolescents? |
|  | 8. This theme included the importance of improving the experience for children and adolescents living with a chronic disease, condition, delay, or disability; health literacy; teamwork; and relationships. |
|  | 17. What factors affect how psoriasis will progress or whether it will go into remission? |
| Prognosis and Disease Course | 18. How do changes in female hormones, such as during puberty, pregnancy, miscarriage, menopause and contraceptive use, affect psoriasis and its treatment? |
|  | 4 At what point in the care pathway (eg, crisis intervention, prevention, engagement, treatment, maintenance, and recovery) are digital interventions most safe and effective? |
|  | 6. How can the course (flares, extensions, cure) of JIA be better explained and predicted? |
|  | 4. What are the short term and long term outcomes of surgery compared to non-surgical care in the treatment of Perthes disease? |
|  | What age is my child likely to live to? |
|  | What is the normal milestone development for a child with the same condition as my child? |
|  | What is the normal milestone development for a child with the same condition as my child? |
|  | Does the disease course of lichen sclerosus differ in boys and girls, adult males and females? |
|  | What factors influence the progression of AMD? |
|  | Can vision be corrected in later life for people with amblyopia? |
|  | What causes keratoconus to progress and can progression be prevented? |
|  | How can glaucoma be stopped from progressing? |
|  | Is there a link between treatment adherence and glaucoma progression and how can adherence be improved? |
|  | Can the diagnosis of inherited retinal diseases be refined so that individuals can be given a clearer idea about their specific condition and how it is likely to progress? |
|  | Why does disease burn out in patients with ocular and orbital inflammatory diseases? |
|  | Does early diagnosis of refractive error improve long-term prognosis and promote faster, more effective treatment? |
| Prognosis and Disease Course | What is the effect of congenital nystagmus on visual and emotional development? |
|  | 2. What are the long term feeding problems in relation to Post Intensive Care Syndrome once a child is discharged home from intensive care? |
|  | 8. Does a child’s swallowing change as a result of having needed a breathing tube |
|  | 10. Do the energy (calorie) needs change for children who have been on a breathing machine for more than 5 d with no change in activity level? |
|  | 3 What are the effects of CHD, low oxygen saturations and interventions on brain development and behavioural outcomes, and how can these be improved? |
|  | 10 What are the long-term outcomes and life expectancy of children born with CHD? |
|  | 10 Does influencing the gut microbiota influence the course of IBD? |
|  | 3 Are there different meaningful types of hyperacusis? |
|  | 5) Assess the effect of mixed tone (spasticity and dystonia) in CP in outcomes and approaches |
|  | 8) Characterize the natural history of DCP |
|  | 8. What is the typical pattern of development of breast feeding in premature babies? |
|  | 1. Pain and fatigue are often present when the disease is in remission. How does this happen, what can one do about it, and can one predict which patients will suffer from them? |
| Potential Interventions | How can better interventions be developed that are effective in treating vitreous opacities/ eye floaters? |
|  | Can a functioning prosthetic eye be developed to replace an eye damaged by injury? |
|  | Can stem cells be used to regrow an eye or part of an eye? |
| Potential Interventions | 6 How can existing cornstarch preparations be modified or alternative treatments be implemented that are easier to administer and/or keep blood sugar levels more stable for patients with liver GSD? |
|  | Can bioengineered organs be developed to be as safe as human-to-human transplants? How can this be achieved? |
|  | 5 Treatment Prospective retinoblastoma treatment studies with long-term follow-up |
|  | 8. What role might tissue engineering have in treating wounds in patients with DEB? |
|  | 9. What role might stem cell therapy and bone marrow transplantation play in treating DEB? |
|  | 10. What role might growth hormone play in decreasing growth delay and puberty delayed in DEB patients? |
|  | 1) Develop new treatments for individuals with DCP |
|  | 15. Could gene therapy help to treat psoriasis? |
|  | 1 What are the benefits and risks of delivering mental health care through technology instead of face-to-face and what impact does the removal of face-to-face human interaction have? |
|  | 3 How can treatment outcomes be maximised by combining existing treatment options (medication, psychological therapies, etc) with digital mental health interventions? |
|  | 5 How should apps for mental health be evaluated and endorsed? |
|  | 6 What impacts will the adoption of digital technology in mental health services have on capacity, access to services, waiting times, and preferred appointment times? |
|  | 7 Are therapies (eg, cognitive behavioural therapy) delivered via digital technology as effective as those delivered face-to-face? |
|  | 8 Can the common elements of therapy (eg, empathy, gestures, non-verbal cues) that come from person-to-person interactions be maintained with digital technology interventions? |
| Potential Interventions | 9 Do digital health interventions increase reach and access to groups and people less well served by traditional mental health services (eg, black and ethnic minorities, men with depression, people in rural areas, etc)? |
|  | 10 How can social media be used more effectively to bring people with mental health problems together and help them connect (eg, in their communities), rather than isolating them in their homes? |
|  | 6. Is urodynamic testing prior to surgery for urinary incontinence associated with better continence rates and quality of life, than surgery indicated without such testing? |
|  | What alternatives to treat cataracts other than cataract surgery are being developed? |
|  | Should accommodative lenses be developed for cataract surgery? |
|  | How can treatment for visual pathway damage associated with preterm birth be developed? |
|  | Can the treatment of amblyopia be improved to produce better short-term and long-term outcomes than are possible with current treatments? |
|  | Can better treatments for glaucoma in children be developed? |
|  | Can a treatment be developed to improve vision for people with albinism? |
|  | Can new therapies such as gene or stem cell treatments be developed for corneal diseases? |
|  | What is the most effective management for dry eye and can new strategies be developed? |
|  | Can treatments to save eye sight from microbial keratitis be improved? |
|  | Can a treatment to slow down progression or reverse sight loss in inherited retinal diseases be developed? |
|  | Can vision loss due to optic nerve diseases such as giant cell arteritis, Leber’s hereditary optic neuropathy, optic neuritis and optic atrophy, be restored, eg, through gene therapy and stem cell treatment? |
| Potential Interventions | Can treatments be developed for visual field and ocular motility manifestations following stroke? |
|  | How can electronic devices improve or restore vision for people with optic neuropathies? |
|  | Can an alternative or new treatment be developed that will treat the sight loss caused by giant cell arteritis? |
|  | Can gene-based targeted therapies for ocular cancers be developed? |
|  | Can adjuvant therapies be developed to treat ocular melanoma? |
|  | Can an effective long lasting treatment for diabetic macular oedema, both ischaemic and non-ischaemic, be developed? |
|  | Can new non-invasive treatments be developed to slow down the progression of diabetic retinopathy? |
|  | 5 How can technology be used to deliver personalised care and improve outcomes in CHD (eg, artificial intelligence, 3D printing, genomics, stem cells, organ regeneration)? |
| Etiology and risk factors | How can the risk of losing sight for people with retinal detachment be reduced? |
|  | What causes posterior vitreous detachment/vitreous syneresis? |
|  | 8. To what extent is psoriasis caused by a person’s genes or other factors. such as stress, gut health. water quality. change in the weather or temperature? |
|  | 6. Which lifestyle factors affect acne susceptibility or acne severity the most and could diet be one of them? |
|  | 1. What are the causes of IBD (Crohn disease, ulcerative colitis)? |
|  | 6 Is hyperacusis due to physical or psychological issues or is it a combination of both? |
|  | 7) Identify what causes DCP (the pathophysiologic mechanism) |
|  | 8. To what extent is psoriasis caused by a person’s genes or other factors, such as stress, gut health, water quality or change in the weather/temperature? |
| Etiology and risk factors  Etiology and risk factors | 13. Are lifestyle factors such as diet, alcohol intake, weight change and smoking involved in causing psoriasis? |
|  | 5. How does JIA develop and which factors influence this? |
|  | What are the genetic and environmental causes of cleft lip with or without cleft palate? |
|  | What is the cause of AMD? |
|  | Can the development of AMD be predicted? |
|  | What is the cause of cataract? |
|  | What are the causes of coloboma and microphthalmia/anophthalmia and how can they be prevented? |
|  | What is the cause of keratoconus and can it be prevented? |
|  | What causes glaucoma? |
|  | What factors affect the progression of sight loss in inherited retinal diseases? |
|  | What causes sight loss in inherited retinal diseases? |
|  | What is the underlying cause of optic nerve damage in optic neuropathies, such as anterior ischaemic optic neuropathy, Leber’s hereditary optic neuropathy, optic neuritis and other optic neuropathies? |
|  | What activates choroidal melanoma metastasis in the liver after the primary melanoma has been treated? |
|  | What are the causes of ocular cancer and how can they be prevented? |
|  | What causes thyroid eye disease? |
|  | What causes birdshot retinopathy? |
|  | What causes scleritis? |
|  | What factors influence the development of refractive error (myopia, astigmatism, presbyopia and long-sightedness)? |
|  | What is the cause of both congenital and acquired nystagmus? |
|  | What are the predictive factors for the progression to sight threatening diabetic eye disease? |
|  | 10) What are the early predictors of ADHD in children? |
| Mental Health | (9) Which strategies are effective in preventing stigma and bullying towards children and young people with learning difficulties? |
|  | 1 What are effective strategies for screening and prevention of mental health problems? |
|  | 9. What is the impact of access to psychological/mental health support in the management of pediatric IBD? |
|  | 9. What is the interaction between chronic pain and mental health symptoms in children and adolescents, and when and how can co-occurring chronic pain and mental health symptoms be most effectively diagnosed and treated? |
|  | 1. What psychological support package improves psychological well-being, social functioning and mental health during and after treatment? |
|  | 6) What mental health supports can be provided to parents, families, and children and youths while hospitalized on the GPIU? |
|  | 2. This theme focused on looking beyond the medical focus of care and how nurses can better decrease stress and improve psychological support for children and families. |
|  | 2. Reducing fear and anxiety in children throughout the hospital experience |
|  | 4. Developing more effective and age-appropriate tools to address children's anxiety. Level of pain and understanding of the hospital process |
|  | 16. How does psoriasis affect a person psychologically? |
|  | 2 How do certain mental health conditions (eg, depression) affect how people engage with technology? |
|  | What is the psychosocial impact of my child’s condition on my child and our family? |
| Mental Health | What is the psychosocial impact of my child’s condition on my child and our family? |
|  | What is the psychosocial impact of my child’s condition on my child and our family? |
|  | What is the psychosocial impact of my child’s condition on my child and our family? |
|  | What is the relationship between sight loss and mental health for people with inherited retinal diseases? |
|  | 3. How should we best manage emotional well-being in children and young people with neurological conditions? |
|  | 14. What are the key issues facing children in terms of their mental health and what can we do about them? |
|  | 15. How does perception of pressure to succeed impact on health and wellbeing? |
|  | 6 What is the impact of living with CHD on mental health in children and how can this be improved through access to psychological support and other therapies? |
|  | 1) What are the best strategies for the prevention of mental health issues in children and families? |
|  | 2) How can parents best support their childres's mental health during times of drastic change? |
|  | 6) How are early childhood behaviours related to a child's future mental health? |
|  | 8) What are the causes of increasing mental illness in children and adults? |
|  | 6) Which challenges of chronic conditions and disabilities impact mental health in young people? |
| Family and Caregiver support | 9 What is the most effective way of educating families and caregivers on anorexia nervosa and on ways to support patients through recovery? |
|  | (6) How can parents, carers, brothers and sisters and extended families of children and young people with learning difficulties, be best supported to achieve their best quality of life before, during and after the diagnosis or identification in home, school and community contexts? |
| Family and Caregiver support | 3 Psychosocial How to provide culturally competent social, emotional and psychological support to patients with retinoblastoma, survivors, parents and families (at diagnosis and beyond)? |
|  | 8. How can parents/carers/siblings/partners be best supported following the death of a young person with cancer? |
|  | 10) Increase awareness of DCP among families |
|  | 2) What methods of communication are most effective between patients, caregivers, and health care providers on a GPIU? |
|  | 7. This theme was about quality of experience for children and their families and how nurses can better provide support for the child and the family |
|  | 8 Improving communication with parents |
|  | Where would I find specialized information such as video clips of parents feeding a baby with my child’s condition? |
|  | What devices or products are the best to buy for my child at different life stages? |
|  | Where would I find specialized information such as video clips of parents feeding a baby with my child’s condition? |
|  | 9. What strategies should be in place on PICU to help with parental bonding with their baby when breast feeding is not possible? |
|  | 4. What are the most effective strategies to support communication in children and young people with neurological conditions? (e.g. use of high and low technology augmentative alternative communication (AAC) and to improve speech intelligibility) |
|  | 12. How do family relationships impact children’s health and wellbeing? |
|  | 13. How can we ensure their voices are heard and can infuence their future? |
| Family and Caregiver support | 18. What is needed to understand how to support or improve parenting skills? |
|  | 23. How do children’s peer and social relationships afect their health and wellbeing? |
|  | 8) How can we promote and respect young people's agency and self‐advocacy? |
|  | 10) How can coordination and communication with young people and their families be improved across and between all disciplines and communityservices? |
|  | 4. What is the psychosocial impact of tube feeding on (a) the carers of premature infants and (b) the bond between carers and premature infants who are tube fed? |
| Health Care professional training | 5 What are the best ways to educate healthcare professionals (e.g., medical students, primary care, mental health specialists, etc.) and educators about anorexia nervosa, and how does this impact early identification rates, access to services, and recovery? |
|  | 4. How can errors and patient injuries in anesthesia care be prevented? |
|  | (1) What knowledge, skills and training do educational professionals need to identify the early signs of learning difficulties and provide optimal support for children and young people affected to help them achieve the best possible outcomes? |
|  | (5) What knowledge, skills and training do health, social work and ‘third sector’ (eg, charities and support services) professionals need to understand the best support to give children and young people with learning difficulties and their families/carers? |
|  | 4 What is the essential knowledge/training required for health professionals to appropriately refer or effectively manage hyperacusis? |
|  | 4 What is the most effective strategy to educate people with asthma and health professionals about managing the adverse effects of drug therapies? (Asthma UK) |
|  | 8 What type of patient (children and adults) and health professional education is most effective in gaining asthma control? (Asthma UK) |
| Health Care professional training | 5. What strategies effectively increase health care providers’ training, knowledge, recognition, beliefs, attitudes and communication about the validity and risk of chronic pain in children and adolescents, and its evidence-based treatments? |
|  | 4. What General Practitioner or young person strategies, such as awareness campaigns and education, improve early diagnosis for young people with suspected cancer? |
|  | 10) What are the most effective communication methods (eg, handover, rounds) between health care providers on a GPIU? |
|  | 3. This theme included clinical handover; consistency; communication techniques; care plans; and communication between different parties such as children and staff, staff and staff, service and service |
|  | 2. Can guidance or training for general practitioners on appropriate pathways of care improve the management of patients with urinary incontinence? |
|  | 9. What are the best strategies to optimise communication of information between patients/carers and clinicians in order to enable shared decision making? |
|  | Are there lactation consultants with expertise in supporting parents who have a child like mine? |
|  | 4. Can parents’ nutritional beliefs and preferences be better understood by healthcare professional and included into the care of their child on breathing machines? |
|  | 9. This theme was about how nurses can create a safe physical, psychosocial and cultural environment for children and their families in the healthcare system. |
|  | 10. What is the clinical and cost effectiveness of speech and language therapists training to improve outcomes for children with cleft palate and eating and drinking difficulties? |
| Comorbidities | 10. What is the best way of eradicating /Pseudomonas aeruginosa/ in people with CF? |
|  | 5. Is there a way of preventing CF related diabetes (CFRD) in people with CF? |
| Comorbidities | 7 What is the most effective way of treating cooccurring/comorbid disorders for individuals with anorexia nervosa, and should treatments occur separately or at the same time? Why? |
|  | 6. Does treating psoriasis help improve other health conditions. such as psoriatic arthritis, cardiovascular disease, metabolic syndrome and stress? |
|  | 9. Is a person with psoriasis more likely to develop other health conditions (either as a consequence of psoriasis or due to the effect of treatments for psoriasis)? If so, which ones? |
|  | 7 Second cancer What are the risk factors for second cancers in heritable retinoblastoma survivors, and, in turn, what do heritable retinoblastoma survivors need to know about living well and minimizing risk of second cancers? |
|  | 2 What is the most effective way of managing asthma with other health problems? (Asthma UK) |
|  | 6. Does treating psoriasis help improve other health conditions, such as psoriatic arthritis, cardiovascular disease, metabolic syndrome and stress? |
|  | 9. Is a person with psoriasis more likely to develop other health conditions (either as a consequence of psoriasis or due to the effect of treatments for psoriasis)? If so, which ones? |
|  | Is obesity a problem with my child’s condition? |
|  | Is obesity a problem with my child’s condition? |
|  | What is the risk of developing cancer in patients with lichen sclerosus? |
|  | What are the outcomes for cataract surgery among people with different levels of cognitive impairment (all causes excluding dementia, stroke, neurological conditions, head injuries)? |
|  | 7. Improving the experience of children with special needs undergoing surgery (e.g., autism spectrum disorder, ADHD) |
| Early Education | 9. Which school characteristics (eg, policies, attitudes of staff, etc) are most effective to promote inclusion of children and young people with neurodisability in education and afterschool clubs? |
|  | (2) What is the best educational and community environment for children and young people with learning difficulties? |
|  | 3 What is the impact of daycare attendance on child health and development? |
|  | 2. What are the effects of teacher support? |
|  | 2. What is the impact of living with chronic pain on children’s and adolescents’ academic performance and educational attainment, and what strategies best support vocational planning for children and adolescents with chronic pain? |
|  | 7. What strategies for educating school personnel about pediatric chronic pain effectively increase their awareness, understanding and recognition of the validity, impact and treatment of pediatric chronic pain? |
|  | How can I maximize my child’s educational attainment? |
|  | How many children, with the same condition as my child, go to mainstream school? |
|  | How can I maximize my child’s educational attainment? |
|  | If my child has to take time out of school, will their education continue? |
|  | How can I maximize my child’s educational attainment? |
|  | How many children, with the same condition as my child, go to mainstream school? |
|  | How can I maximize my child’s educational attainment? |
|  | 6. How does children’s educational experience impact on their health and wellbeing? |
| Quality of Life | (10) Which strategies are effective in helping children and young people with learning difficulties live independent lives, including during times of transition? |
|  | 4. How much does management in reference centers help patients with DEB (in terms of quality of life, avoiding complications and disability, costeffectiveness)? |
|  | 1. What is the influence of JIA on future opportunities regarding school results, work, and relationships? |
|  | 7. What interventions are most effective in supporting young people when returning to education or work? |
|  | What is the impact on quality of life? |
|  | Would correction of refractive error have a positive impact on early life learning and development? |
|  | 7 What is the impact of living with CHD on quality of life in children and how can this be improved? |
|  | 5. What are the best ways of supporting a young person who has incurable cancer? |
|  | 2. Why are children with JIA fatigued more quickly, what can be done about it, and how can one cope with the fatigue in daily life? |
|  | 2. Why are children with JIA fatigued more quickly, what can be done about it, and how can one cope with the fatigue in daily life? |
|  | 10. What is the best way to treat sudden flare-ups of psoriasis? |
|  | What can be done to help ocular cancer sufferers? |
| Integrated Knowledge Translation | 2. What are the impacts of involving patients in shared decision-making about anesthesia and care options before, during, and after surgery? |
|  | 3. What data should be collected from patients about anesthesia care before, during, and after surgery to better understand their outcomes and experiences? |
|  | 8. How can patients’ feedback about their experiences before, during, and after surgery be used to improve anesthesia care? |
| Integrated Knowledge Translation | 7) What are effective ways to incorporate shared decision-making with parents and children/youths hospitalized on the GPIU? (effectiveness defined as length of stay, caregiver confidence) |
|  | 6. This theme included that research was needed around and in collaboration with families related to their culture, disability, family structures, and geographic location. Social determinants of health; use of interpreters; and refugees were also highlighted. |
|  | 10. This included research on neonatal care and psychosocial needs of infants in the first 1000 days. |
|  | What is the latest genetic research relating to my child’s condition? |
|  | What is the latest genetic research relating to my child’s condition? |
|  | 2 Can consensus guidelines (for management) be achieved for patients with liver GSD? |
|  | 3. What is the short-term and long-term clinical and cost effectiveness of orthopaedic lower limb surgery for children with Cerebral Palsy who can walk (considering best timing and technique)? |
|  | What are the most effective treatments and rehabilitation for optic neuropathies, eg, Leber’s hereditary optic neuropathy and anterior ischaemic optic neuropathy? |
| Care coordination & health system navigation | (3) How can multiple types of professionals work together with parents and carers to improve identification, diagnosis, interventions and treatments and achieve the best outcomes for children and young people with learning difficulties? |
|  | 4 Follow-up What is the optimal follow-up (including ophthalmologic and oncologic) for patients with heritable retinoblastoma and survivors (by diagnosis and treatment), and how can we ensure this is provided to all? |
|  | 9 Follow-up How to provide a detailed pathway of care or plan, outlining treatment and follow-up, to patients with retinoblastoma and families? |
|  | 9 What is the most effective way to manage consultations and asthma control in adolescence and young people? (Asthma UK) |
| Care coordination & health system navigation | 9. What is the best method of follow-up and timing which causes the least psychological and physical harm, while ensuring relapse/ complications are detected early? |
|  | 1) What best practices and/or care models exist for inpatient care for children and youths with medical complexity on the GPIU? |
|  | 9) What are effective alternatives to shorten length of stay for hospitalized children and youths on the GPIU? (eg, hospitalization at home, early discharge with close and regular follow-up) |
|  | 1. This theme included issues such as timely access to service; appropriate local service; referral pathways; updated resources, programs, and booking systems; and reducing time out of school for attending appointments. |
|  | 4. This theme reflected transition from pediatric to adult services; from hospital to home; from tertiary to regional services; as well as support to new parents. |
| Treatment effect modifiers | 3. What factors predict how well psoriasis will respond to a treatment? |
|  | 1. Which factors before, during, and after receiving anesthesia for surgery are most important to improve patient outcomes and satisfaction? |
|  | 1 What is the optimal treatment strategy considering efficacy, safety and cost-effectiveness (immunomodulators, biologics, surgery, combinations) in IBD management: selecting the right patient group, right stage of disease, and assessing potential for withdrawal? |
|  | 5 What is an optimal treatment strategy for perianal Crohn’s disease and what individual factors determine this? |
|  | 6) Assess predictors of treatment responsiveness (e.g. etiology, severity, earlier detection) in individuals with DCP |
|  | 3. What factors predict how well psoriasis will respond to a treatment? |
|  | 7. Why do psoriasis treatments stop working well against psoriasis and when they stop working well, what’s the best way to regain control of the disease? |
| Treatment effect modifiers | 2. What is the best treatment plan for each individual patient? (e.g. start a biological directly, which one, and what to do when the first one does not work and how can medication best be tapered off? |
|  | 3. What is the best treatment plan for uveitis in JIA, and are there factors that predict its effectiveness? |
