# Supplementary material for: Identifying Common Patient‐Oriented Priorities for Child and Adolescent Health Research and Care: A Systematic Review of Priority Setting Partnerships
Source: Health Expect. 2025 Jul 30;28(4):e70349. doi: 10.1111/hex.70349 (PMC12309730; doi:10.1111/hex.70349)
Supplement: Supplementary file 4 — Supplemental Table S2: JLA Priority Setting Partnership Study Characteristics. [file HEX-28-e70349-s003.docx]

**Supplemental Table S2 –** JLA Priority Setting Partnership Study Characteristics

| **Lead Author** | **Date Published** | **Population/Topic of Focus** | **Country** | **Funders/Organizations Involved** | **Sample Size** | **Uncertainties** |
| --- | --- | --- | --- | --- | --- | --- |
| Finlay-Jones et al. ^47^ | August 2023 | Children and young people with chronic conditions and disabilities | Australia | Perth Children's Hospital Foundation; Medical School, University of Western Australia*; School of Population Health, Curtin University*;  Telethon Kids Institute, Perth Children's Hospital* | *Initial survey* Children/adolescents/people with lived experience: n=94 (47%) Parents/Caregivers/Family members: n=78 (39%) Clinicians/Healthcare professionals: n=28 (14%)  *Interim survey* Children/adolescents/people with lived experience: n=75 (36%) Parents/Caregivers/Family members: n=95 (47%) Clinicians/Healthcare professionals: n=33 (16%)  *Workshop* Children/adolescents/people with lived experience: n=12 (57%) Parents/Caregivers/Family members: n=3 (14%) Clinicians/Healthcare professionals: n=6 (29%) | Initial: 456 Final: 10 |
| Cartwright et al.^48^ | April  2023 | Priorities for happy and healthy children in community (Bradford) | UK | Born in Bradford, Braford Institute for Health Research, Bradford Teaching Hospitals NHS Foundation Trust*;  National Institute for Health and Care Resarch (NIHR) Applied Research Collaboration (ARC) Yorkshire and Humber;  ActEarly UK Prevention Research Partnership | *Initial survey* Children/adolescents/people with lived experience: n=22 (4%)  Parents/Caregivers/Family members: n=296 (60%) Public and professionals working with children: n=145 (29%) Researchers: n=29 (6%)  *Interim survey* Unspecified  *Workshop: n=14* Unspecified | Initial: 5748 Final: 27 |
| Sommerfield et al.^49^ | February 2023 | Pediatric Anesthesia and perioperative medicine | Australia | Perth Children's Hospital, Queensland Children's Hospital and the Women and Children's Hospital in Adelaide*, Perth Children's Hospital Anesthesia Research Consumer Reference Panel* | *Initial survey* Parents/Caregivers/Family members: n=356 (83%) Clinicians/Healthcare professionals: n=75 (17%)  *Interim survey* Parents/Caregivers/Family members: n=566 (52%) Clinicians/Healthcare professionals: n=525 (48%)  *Workshop* Parents/Caregivers/Family members: n=10 (59%) Clinicians/Healthcare professionals: n=7 (41%) | Initial: 281 Final: 10 |
| Eaton et al.^50^ | January 2023 | Child and family health^b^ | Canada | Department of Pediatrics, Faculty of Medicine and Dentistry, University of Alberta*; Women and Children’s Health Research Institute (WCHRI) at the University of Alberta | *Initial survey* Parents/Caregivers/Family members: n=100 (80%) Clinicians/Healthcare professionals: n=25 (20%)  *Interim survey* Parents/Caregivers/Family members: n=100 (80%) Clinicians/Healthcare professionals: n=25 (20%)  *Workshop* Parents/Caregivers/Family members: n=7 (58%) Clinicians/Healthcare professionals: n=5 (42%) | Initial: 389 Final: 10 |
| British Paediatric Neurology Association^23 f^ | November 2022 | Childhood Neurological Disorders | UK | Neurosciences Programme, University College London, Institute of Child Health*^a^;  Evelina London Hospital, Guy’s and St Thomas’ NHS Foundation Trust*; British Paediatric Neurology Association; | *Initial survey* Children/adolescents/people with lived experience: n=62 (9%) Parents/Caregivers/Family members: n=382 (53%) Clinicians/Healthcare professionals: n=221 (30%) Other professional: n=30 (4%) Other persons affected: n=31 (4%)  *Interim survey* Children/adolescents/people with lived experience: n=293 (18%) Parents/Caregivers/Family members: n=791 (49%) Clinicians/Healthcare professionals: n=93 (6%) Other professional: n=299 (18%) Other persons affected: n=148 (9%)  *Workshop* Children/adolescents/people with lived experience: n=2 (7%) Parents/Caregivers/Family members: n=11 (41%) Clinicians/Healthcare professionals: n=14 (52%) | Initial: 297 Final: 10 |
| Drury et al.^27^ | November 2022 | Congenital heart disease | UK | University of Birmingham*^a^; British Heart Foundation;  George Davies Charitable Trust to the Birmingham Children's Hospital Charity | *Initial survey* Children/adolescents/people with lived experience: n=133 (25%) Parents/Caregivers/Family members: n=247 (47%) Clinicians/Healthcare professionals: n=135 (26%) Other family members: n=7 (1%) Unknown: n=2 (0%)  *Interim survey* Children/adolescents/people with lived experience: n=36 (14%) Parents/Caregivers/Family members: n=105 (42%) Clinicians/Healthcare professionals: n=101 (40%) Other family members: n=5 (2%) Charities: n=3 (1%)  *Workshop* Children/adolescents/people with lived experience: n=9 (23%) Parents/Caregivers/Family members: n=8 (20%) Clinicians/Healthcare professionals: n=21 (54%) Charities: n=1 (3%) | Initial: 1060 Final 10^c^ |
| Gilbert et al.^51^ | August 2022 | Dystonia in cerebral palsy patients | USA | Washington University School of Medicine*; St. Louis Children's Hospital* | *Initial survey* Children/adolescents/people with lived experience: n=5 (18%) Parents/Caregivers/Family members: n=11 (39%) Clinicians/Healthcare professionals: n=11 (39%) CP Community Advocate: n=1 (4%)  *Interim survey* Children/adolescents/people with lived experience: n=10 (12%) Parents/Caregivers/Family members: n=24 (29%) Clinicians/Healthcare professionals: n=46 (56%) CP Community Advocate: n=2 (3%)  *Workshop* Clinicians/Healthcare professionals: n=3 (60%) CP Community Members: n=2 (40%) | Initial: 67 Final: 10 |
| Vera San Juan et al.^52^ | May  2022 | Adolescent mental health | UK | National Institute for Health and Care Research (NIHR)* | *Initial survey* Clinicians/Healthcare professionals: n=9 (100%)  *Interim survey* Children/adolescents/people with lived experience: n=26 (64%) Parents/Caregivers/Family members: n=127 (36%)  *Workshop survey* Children/adolescents/people with lived experience: n=46 (68%) Parents/Caregivers/Family members: n=15 (22%) Teachers: n=7 (10%) | Initial: 66 Final: 4 |
| Gill et al.^53^ | April  2022 | Pediatric hospital inpatients | Canada | Department of Pediatrics, University of Toronto*^a^; Division of Paediatric Medicine, The Hospital for Sick Children*; Canadian Institutes of Health Research (CIHR); | *Initial survey* Children/adolescents/people with lived experience: n=9 (5%) Parents/Caregivers/Family members: n=41 (22%) Clinicians/Healthcare professionals: n=125 (66%) Friend/Family Member: n=4 (2%) Other: n=9 (5%)  *Interim survey* Children/adolescents/people with lived experience: n=10 (5%) Parents/Caregivers/Family members: n=33 (16%) Clinicians/Healthcare professionals: n=136 (68%) Friend/Family Member: n=1 (1%) Other: n=21 (10%)  *Workshop* Children/adolescents/people with lived experience: n=2 (8%) Parents/Caregivers/Family members: n=10 (42%) Clinicians/Healthcare professionals: n=12 (50%) | Initial: 437 Final: 10 |
| O'Connor et al.^54 e^ | March 2022 | PICU nutrition | UK | Department of Dietetics, Great Ormond Street Hospital*^a^; The British Dietetic Association | *Initial survey: n=90* Parents/Caregivers/Family members: 12% Clinicians/Healthcare professionals: 88%   *Interim survey:* *n=140* Children/adolescents/people with lived experience: 4% Parents/Caregivers/Family members: 14% Clinicians/Healthcare professionals: 82%  *Workshop* Parents/Caregivers/Family members: n=9 (64%) Clinicians/Healthcare professionals: n=5 (36%) | Initial: 165 Final: 10 |
| Pagnamenta et al.^28^ | January 2022 | Dysphagia | UK | Royal College of Speech and Language Therapists*^a^; National Institute for Health and Care Research (NIHR)* | *Initial survey* Clinicians/Healthcare professionals: n=156 (100%)  *Interim survey* Children/adolescents/people with lived experience: n=4 (1%) Parents/Caregivers/Family members: n=7 (2%) Clinicians/Healthcare professionals: n=367 (95%) Patient Organization: n=9 (2%)  *Workshop* Children/adolescents/people with lived experience: n=4 (11%) Parents/Caregivers/Family members: n=1 (3%) Clinicians/Healthcare professionals: n=18 (49%) Clinical academics/researchers: n=10 (27%) Patient Organization: n=1 (3%) NIHR Research Facilitators: n=3 (8%) | Initial: 111 Final 10^c^ |
| Mörelius et al.^55^ | December 2021 | Pediatric and child health nursing | Australia | Child and Adolescent Health Service* | *Initial survey* Children/adolescents/people with lived experience: 9 (35%) Parents/Caregivers/Family members: 8 (31%) Clinicians/Healthcare professionals: 9 (35%)  *Interim survey* Children/adolescents/people with lived experience: 8 (3%) Parents/Caregivers/Family members: 86 (37%) Clinicians/Healthcare professionals: 135 (58%) Other: 3 (1%)  *Workshop* Children/adolescents/people with lived experience: 4 (21%) Parents/Caregivers/Family members: 8 (42%) Clinicians/Healthcare professionals: 7 (37%) | Initial: 23 Final 10 |
| Aussems et al.^56^ | August 2021 | Juvenile idiopathic arthritis | The Netherlands | Amsterdam UMC*^a^; Dutch Association for Paediatric Rheumatology;  Dutch JIA Patient and Parent Organisation;  Wilhelmina Children's Hospital; PGOsupport | *Initial survey: n=42* Children/adolescents/people with lived experience Researchers  *Interim survey: n=42* Children/adolescents/people with lived experience  *Workshop* Children/adolescents/people with lived experience Researchers | Initial: 604 Final: 5 |
| Verwoerd et al.^57^ | April 2021 | Juvenile idiopathic arthritis | The Netherlands | Dutch JIA patient and parent organisation (JVN)*^a^;  Youth-R-Well.com (YRW)*;  Dutch Society for Paediatric Rheumatology (NVKR)*;  Dutch Health Professionals in Paediatric Rheumatology (NHPKR)*;  Centre for Translational Immunology, University Medical Centre Utrecht;  Department of Paediatric Immunology and Rheumatology, Wilhelmina Children’s Hospital | *Initial survey* Children/adolescents/people with lived experience: n=141 (51%) Parents/Caregivers/Family members: n=88 (32%) Clinicians/Healthcare professionals: n=49 (18%)  *Interim survey* Children/adolescents/people with lived experience: n=125 (41%) Parents/Caregivers/Family members: n=136 (45%) Clinicians/Healthcare professionals: n=42 (14%)  *Workshop* Children/adolescents/people with lived experience: 6 (29%) Parents/Caregivers/Family members: n=5 (24%) Clinicians/Healthcare professionals: n=10 (48%) | Initial: 604 Final: 10 |
| Ismail et al.^58^ | March 2021 | Psoriasis^b^ | UK | Department of Dermatology, Salford Royal Hospital*^a^; The Psoriasis Association | *Initial survey* Children/adolescents/people with lived experience: n=575 (71%) Parents/Caregivers/Family members: n=34 (4%) Clinicians/Healthcare professionals: n=176 (22%) Other: 20 (2%)  *Interim survey* ^d^ Children/adolescents/people with lived experience: n=753 (69%) Parents/Caregivers/Family members: n=74 (7%) Clinicians/Healthcare professionals: n=270 (25%) Other: n=37 (3%)  *Workshop* Children/adolescents/people with lived experience: n=12 (46%) Clinicians/Healthcare professionals: n=11 (42%) Psoriasis Association: n=3 (12%) | Initial: 55 Final: 20 |
| Flegg et al.^59^ | June 2020 | Retinoblastoma | Canada | Department of Ophthalmology and Vision Sciences, The Hospital for Sick Children*; Institute of Cancer Research, Canadian Institutes of Health Research (CIHR);  SickKids Garron Family Cancer Center Conference Fund | *Initial survey* Children/adolescents/people with lived experience: n=10 (17%) Parents/Caregivers/Family members: n=28 (47%) Clinicians/Healthcare professionals: n=16 (27%) Clinician-Scientists: n=3 (5%) Researchers: n=2 (3%)  *Interim survey* Clinicians/Healthcare professionals  *Workshop* Children/adolescents/people with lived experience: n=10 (50%) Non-patients: n=10 (50%) | Initial: 175 Final: 10 |
| McKeen et al.^60^ | June 2020 | Anesthesia | Canada | Department of Anesthesia Perioperative and Pain Medicine, Dalhousie University*^a^; Canadian Institutes of Health Research (CIHR);  Association of University Departments of Anesthesia (ACUDA); Canadian Anesthesiologists’ Society (CAS); Northern Ontario School of Medicine Department of Anesthesia;  Perioperative Anesthesia Clinical Trials Group (PACT);  University of Manitoba Department of Anesthesia | *Initial survey* ^d^ Children/adolescents/people with lived experience: n=114 (45%) Parents/Caregivers/Family members: n=72 (28%) Clinicians/Healthcare professionals: n=180 (71%) Knew someone who got surgery: n=75 (30%)  *Interim survey* Children/adolescents/people with lived experience: n=50 (21%) Parents/Caregivers/Family members: n=18 (8%) Clinicians/Healthcare professionals: n=158 (68%) Knew someone who got surgery: n=7 (3%)  *Workshop* Patients/carers: n=14 (64%) Unspecified: n=8 (36%) | Initial: 574 Final: 10 |
| Obeid et al.^61^ | May 2020 | Anorexia nervosa ^b^ | Canada | Children's Hospital of Eastern Ontario Research Institute*^a^; Canadian Institutes of Health Research (CIHR); | *Initial survey* Children/adolescents/people with lived experience: n=33 (22%) Parents/Caregivers/Family members: n=35 (24%) Clinicians/Healthcare professionals: n=26 (18%) Lived experience and health professional: n=12 (8%) Carer and health professional: n=6 (4%) Lived experience and carer: n=10 (7%) Unspecified: n=23 (16%)  *Interim survey: n=48* Children/adolescents/people with lived experience Parents/Caregivers/Family members Clinicians/Healthcare professionals Other  *Workshop* Children/adolescents/people with lived experience: n=9 (32%) Parents/Caregivers/Family members: n=9 (32%) Clinicians/Healthcare professionals: n=8 (29%) Lived experience and healthcare professional: n=2 (7%) | Initial: 897 Final: 10 |
| Peeks et al.^62^ | March 2020 | Liver glycogen storage diseases (GSD) | International (58 countries), based in the Netherlands | University Medical Center Groningen, University of Groningen*^a^;  International GSD Conference held at the University Medical Centre Groningen;  Scandinavian Association for Glycogen Storage Disease;  Selbsthilfegruppe Glykogenose Deutschland e.V. | *Initial survey*  ^d^ Children/adolescents/people with lived experience: n=150 (20%) Parents/Caregivers/Family members: n=370 (48%) Clinicians/Healthcare professionals: n=266 (35%) Unspecified: n=26 (3%)  *Interim survey* Children/adolescents/people with lived experience: n=86 (15%) Parents/Caregivers/Family members: n=253 (45%) Clinicians/Healthcare professionals: 166 (30%) Unspecified: 57 (10%)  *Workshop* Children/adolescents/people with lived experience: n=5 (18%) Parents/Caregivers/Family members: n=12 (43%) Clinicians/Healthcare professionals: n=11 (39%) | Initial: 1388 Final: 11 |
| Vella-Baldacchino et al.^63^ | December 2019 | Children requiring elective surgery for conditions affecting the lower limbs | UK | Botnar Research Centre*^a^;  Oxford Biomedical Research Centre;  The British Society of Children’s Orthopaedic Surgery;  The British Orthopaedic Association; James Lind Alliance | *Initial survey: n=388* Patients, parents or carers: 47% Clinicians/Healthcare professionals: 53%  *Interim survey* Patients, parents or carers: n=117 (50%) Clinicians/Healthcare professionals: n=117 (50%)  *Workshop* Children/adolescents/people with lived experience: n=4 (13%) Parents/Caregivers/Family members: n=9 (30%) Clinicians/Healthcare professionals: n=14 (47%) Charity representatives: n=2 (7%) Clinical scientists: n=1 (3%) | Initial: 75 Final: 10 |
| Birnie et al.^64^ | November 2019 | Pediatric chronic pain | Canada | University of Toronto*; The Hospital for Sick Children*; Canadian Institutes of Health Research (CIHR);  Alberta Children’s Hospital Research Institute;  Saskatchewan Health Research Foundation;  Children’s Hospital of Eastern Ontario Research Institute; Dalhousie University Faculty of Medicine;  Chronic Pain Network | *Initial survey* Children/adolescents/people with lived experience: n=86 (40%) Parents/Caregivers/Family members: n=50 (23%) Clinicians/Healthcare professionals: n=73 (34%) Other family member: n=6 (3%)  *Interim survey* Children/adolescents/people with lived experience: n=19 (33%) Parents/Caregivers/Family members: n=16 (28%) Clinicians/Healthcare professionals: n=21 (37%) Other family member: n=1 (2%)  *Workshop* Children/adolescents/people with lived experience: n=6 (30%) Parents/Caregivers/Family members: n=5 (25%) Clinicians/Healthcare professionals: n=8 (40%) Other family member: n=1 (5%) | Initial: 540 Final: 10 |
| Sinclair et al.^26^ | November 2019 | Children with Down Syndrome, cleft lip with or without cleft palate, congenital heart defects, or spina bifida | Croatia, France, Germany, Italy, Netherlands, Poland, Portugal, Spain, and the United Kingdom | Institute of Nursing and Health Research, Ulster University*; European Union’s Horizon 2020 | *Initial survey* Parents/Caregivers/Family members: n=32 (100%)  *Interim survey* Parents/Caregivers/Family members: n=80 (100%)  *Workshop* Parents/Caregivers/Family members | Initial: 98 Final: 10^c^ |
| Lim et al.^65^ | October 2019 | Learning difficulties | Scotland | The Salvesen Mindroom Centre*^a^ | *Initial survey* Patients or family members: n=29 (8%) Parents/Caregivers/Family members: n=147 (40%) Clinicians/Healthcare professionals: n=191 (52%)  *Interim survey* Patients or family members: n=41 (11%) Parents/Caregivers/Family members: n=125 (35%) Clinicians/Healthcare professionals: n=195 (54%)  *Workshop* Children/adolescents/people with lived experience: n=5 (20%) Parents/Caregivers/Family members: n=6 (24%) Clinicians/Healthcare professionals: n=5 (20%) Teachers and educational consultants: n=9 (36%) | Initial: 828 Final: 10 |
| Grant et al.^66^ | September 2019 | Pediatric inflammatory bowel disease (IBD) | Canada | Maritime Intestinal Research Alliance (MIRA)*; Canadian Institutes of Health Research (CIHR) | *Initial survey* Children/adolescents/people with lived experience: n=111 (30%) Parents/Caregivers/Family members: n=133 (37%) Clinicians/Healthcare professionals: n=46 (12%) Other/did not indicate: n=73 (20%)  *Interim survey* Children/adolescents/people with lived experience: n=25 (34%) Parents/Caregivers/Family members: n=20 (27%) Clinicians/Healthcare professionals: n=30 (39%)  *Workshop* Children/adolescents/people with lived experience: n=2 (17%) Parents/Caregivers/Family members: n=4 (33%) Clinicians/Healthcare professionals: n=5 (42%) Siblings: n=1 (8%) | Initial: 1209 Final: 10 |
| Simpson et al.^24^ ^e,f^ | May 2019 | Lichen sclerosus ^b^ | International (based out of UK) | University of Nottingham*^a^;  British Society for the Study of Vulval Disease;  James Lind Alliance | *Initial survey* Children/adolescents/people with lived experience: n=383 (59%) Parents/Caregivers/Family members: n=21 (3%) Clinicians/Healthcare professionals: n=222 (34%) Not specified: n=27 (4%)  *Interim survey* ^d^ Children/adolescents/people with lived experience: n=581 (61%) Parents/Caregivers/Family members: n=44 (46%) Clinicians/Healthcare professionals: n=334 (35%) Other n=44 (46%)  *Workshop* Children/adolescents/people with lived experience: n=12 (41%) Parents/Caregivers/Family members: n=2 (7%) Clinicians/Healthcare professionals: n=15 (52%) | Initial: 2580 Final: 10 |
| Aldiss et al.^67^ | November 2018 | Young people with cancer | UK | University of Surrey*^a^;  University College London Hospitals NHS Foundation Trust*; National Cancer Research Institute*; Teenage Cancer Trust;  CLIC Sargent Cancer Care;  Children with Cancer UK; | *Initial survey* Children/adolescents/people with lived experience: n=108 (36%) Parents/Caregivers/Family members: n=101 (34%) Clinicians/Healthcare professionals: n=83 (30%)  *Interim survey* Children/adolescents/people with lived experience: n=58 (33%) Parents/Caregivers/Family members: n=45 (26%) Clinicians/Healthcare professionals: n=71 (41%)  *Workshop* Children/adolescents/people with lived experience: n=7 (28%) Parents/Caregivers/Family members: n=4 (16%) Clinicians/Healthcare professionals: n=14 (56%) | Initial: 855 Final: 10 |
| McPin Foundation ^22 e,f^ | November 2018 | Mental health in children and young people | UK | McPin Foundation*^a^; Economic and Social Research Council; Medical Research Council; Wellcome Trust;  MQ: Transforming Mental Health; Mental Health Research UK | *Initial survey: n=2566* Children/adolescents/people with lived experience: 40% Parents/Caregivers/Family members: 40% Psychologists or psychiatrists: 4% Mental health nurses, therapists, or other support roles: 9% Teachers or school-based support staff: 12%  *Interim survey: n=753* Children/adolescents/people with lived experience Parents/Caregivers/Family members Clinicians/Healthcare professionals Other  *Workshop* Children/adolescents/people with lived experience Parents/Caregivers/Family members Clinicians/Healthcare professionals Other | Initial: 91 Final: 10 |
| Moorhead et al.^68^ | November 2018 | Psoriasis ^b^ | UK | Centre for Dermatology, Manchester Academic Health Science Centre, University of Manchester*^a^;  Salford Royal Hospital*;  Psoriasis Association; | *Initial survey: n=805* Children/adolescents/people with lived experience: 71% Clinicians/Healthcare professionals: 22% Others: 6%  *Interim survey:* *n=1154* Unspecified  *Workshop: n=26* Children/adolescents/people with lived experience: 58% Clinicians/Healthcare professionals: 42% | Initial: 2133 Final: 10 |
| Hollis et al. ^69^ | October 2018 | Digital technology in mental health care^b^ | UK | Institute of Mental Health, University of Nottingham*^a^; National Institute for Health and Care Research (NIHR);  McPin Foundation;  MQ: Transforming Mental Health; | *Initial survey* ^d^ Children/adolescents/people with lived experience: n=353 (57%) Parents/Caregivers/Family members: n=163 (26%) Clinicians/Healthcare professionals: n=365 (59%)  *Interim survey* ^d^ Children/adolescents/people with lived experience: n=357 (58%) Parents/Caregivers/Family members: n=157 (26%) Clinicians/Healthcare professionals: n=345 (56%)  *Workshop* ^d^ Children/adolescents/people with lived experience: n=14 (52%) Parents/Caregivers/Family members: n=3 (11%) Clinicians/Healthcare professionals: n=15 (56%) | Initial: 134 Final: 10 |
| Rowbotham et al.^70^ | April 2018 | Cystic fibrosis ^b^ | International (mostly UK, USA and Canada, Europe, Australia and NZ) | University of Nottingham*; Nottingham Hospitals Charity;  CF Trust Venture and Innovation Award | *Initial survey* Children/adolescents/people with lived experience: n=95 (20%) Parents/Caregivers/Family members: n=105 (22%) Clinicians/Healthcare professionals: n=211 (44%) Family and friends: n=24 (5%) Unspecified: n=47 (10%)  *Interim survey* Children/adolescents/people with lived experience: n=121 (18%) Parents/Caregivers/Family members: n=160 (24%) Clinicians/Healthcare professionals: n=303 (45%) Family and friends: n=33 (5%) Unspecified: n=60 (9%)  *Workshop* Children/adolescents/people with lived experience Parents/Caregivers/Family members Clinicians/Healthcare professionals | Initial: 1080 Final: 10 |
| Lomer et al.^71^ | December 2017 | Inflammatory bowel disease ^b^ | UK | Diabetes & Nutritional Sciences Division, Kings College London*^a^;  Department of Nutrition and Dietetics, Guy’s and St Thomas’ NHS Foundation Trust*;  British Society of Gastroenterology;  Crohn’s and Colitis UK | *Initial survey*: *n=531* Children/adolescents/people with lived experience Clinicians/Healthcare professionals  *Interim survey* Children/adolescents/people with lived experience Parents/Caregivers/Family members Clinicians/Healthcare professionals  *Workshop* Children/adolescents/people with lived experience Parents/Caregivers/Family members Clinicians/Healthcare professionals | Initial: 1671 Final: 10 |
| Lavigne et al.^72^ | August 2017 | Pediatric preventative care research | Canada | North York General Hospital*; Department of Pediatrics | *Initial survey* Parents/Caregivers/Family members: n=115 (73%) Clinicians/Healthcare professionals: n=42 (27%)  *Interim survey* Parents/Caregivers/Family members: n=5 (50%) Clinicians/Healthcare professionals: n=5 (50%)  *Workshop* Parents/Caregivers/Family members: n=10 (36%) Clinicians/Healthcare professionals: n=18 (64%) | Initial: 1046 Final: 10 |
| Knight et al.^73^ | October 2016 | Kidney transplant ^b^ | UK | Centre for Evidence in Transplantation, Clinical Effectiveness Unit, Royal College of Surgeons of England*;  Nuffield Department of Surgical Sciences, University of Oxford*;  National Institute for Health and Care Research (NIHR) Oxford Biomedical Research Centre | *Initial survey* Children/adolescents/people with lived experience: n=69 (38%) Parents/Caregivers/Family members: n=4 (2%) Clinicians/Healthcare professionals: n=66 (36%) Live donors: n=40 (22%) Unspecified: n=4 (2%)  *Interim survey* Children/adolescents/people with lived experience: n=78 (30%) Parents/Caregivers/Family members: n=11 (4%) Clinicians/Healthcare professionals: n=139 (54%) Live donors: n=28 (11%)  *Workshop* Children/adolescents/people with lived experience Parents/Caregivers/Family members Clinicians/Healthcare professionals | Initial: 487 Final: 10 |
| Jacobson et al.^74^ | August 2016 | Attention deficit/hyperactivity disorder (ADHD) | Sweden | Swedish Agency for Health Technology Assessment and Assessment of Social Services (SBU)* | *Initial survey* Children/adolescents/people with lived experience: n=7 (50%) Clinicians/Healthcare professionals: n=7 (50%)  *Interim survey* Children/adolescents/people with lived experience: n=7 (50%) Clinicians/Healthcare professionals: n=7 (50%)  *Workshop* Children/adolescents/people with lived experience: n=6 (46%) Clinicians/Healthcare professionals: n=7 (54%) | Initial: 39 Final: 10 |
| Layton et al.^75^ | July 2015 | Acne | UK with international participation | Department of Dermatology, Harrogate and District NHS Foundation Trust*^a^; UK Dermatology Clinical Trials Network;  Society for Academic Primary Care | *Initial survey* Patients or family members: n=1456 (65%) Parents/Caregivers/Family members: n=132 (6%) Clinicians/Healthcare professionals: n=652 (29%)  *Interim survey* Children/adolescents/people with lived experience: n=1573 (56%) Parents/Caregivers/Family members: n=237 (8%) Clinicians/Healthcare professionals: n=1012 (36%)  *Workshop* Children/adolescents/people with lived experience: n=13 (34%) Clinicians/Healthcare professionals: n=12 (32%) Observers: n=13 (34%) | Initial: 8276 Final: 10 |
| Morris et al.^76^ | January 2015 | Children and young people with neurodisability | UK | PenCRU, University of Exeter Medical School, University of Exeter*^a^;  Paul Polani Fund, Royal College of Paediatrics and Child Health (RCPCH);  British Academy of Childhood Disability | *Initial survey* Children/adolescents/people with lived experience: n=7 (2%) Parents/Caregivers/Family members: n=138 (39%) Clinicians/Healthcare professionals: n=198 (56%) Part of org supporting disabled people: n=2 (0.6%) Academic/researcher: n=2 (0.6%) Teacher: n=2 (0.6%) Unspecified: n=2 (0.6%)  *Interim survey* Children/adolescents/people with lived experience: n=1 (1%) Parents/Caregivers/Family members: n=26 (34%) Clinicians/Healthcare professionals: n=39 (51%) Representatives from charities: n=10 (13%)  *Workshop* Children/adolescents/people with lived experience: n=3 (14%) Parents/Caregivers/Family members: n=7 (32%) Clinicians/Healthcare professionals: n=8 (36%) Representatives from charities: n=3 (14%) Disability advisor: n=1 (4%) | Initial: 809 Final: 10 |
| Rowe et al.^25^ | July 2014 | Sight loss and vision | UK | Fight for Sight*^a^;  NIHR Moorfields BRC;  Royal National Institute of Blind People; Royal College of Ophthalmologists and UK Vision Strategy | *Initial survey: n=2220* Children/adolescents/people with lived experience: Over 65% Partners, relatives, and carers: 12% Clinicians/Healthcare professionals: 17% Parents: 2% Organizations: 6%  *Interim survey* Patients, carers, and relatives: n=446 (67%) Clinicians/Healthcare professionals: n=218 (33%)  *Workshop* Children/adolescents/people with lived experience: n=77 (50%) Clinicians/Healthcare professionals: n=78 (50%) | Initial: 4461 Final: 10 |
| Davila-Seijo et al.^77^ | April 2013 | Dystrophic epidermolysis bullosa (DEB) ^b^ | Spain | Department of Dermatology, Complexo Hospitalario de Pontevedra (CHOP)*^a^; Fundación IDI CHOP;  Fundación para la Investigación Biomédica del Hospital Infantil del Niño Jesús;  DEBRA Spain | *Initial survey* Children/adolescents/people with lived experience: n=23 (40%) Parents/Caregivers/Family members: n=18 (31%) Clinicians/Healthcare professionals: n=17 (29%)  *Interim survey* Children/adolescents/people with lived experience: n=13 (28%) Parents/Caregivers/Family members: n=15 (33%) Clinicians/Healthcare professionals: n=18 (39%)  *Workshop* Children/adolescents/people with lived experience: n=3 (20%) Parents/Caregivers/Family members: n=2 (13%) Clinicians/Healthcare professionals: n=6 (40%) DEBRA Spain representative: n=1 (7%) Facilitators: n=3 (20%) | Initial: 10 Final: 6 |
| Batchelor et al.^78^ ^e^ | March 2013 | Eczema^b^ | UK | Centre of Evidence-Based Dermatology, University of Nottingham*^a^; National Institute for Health Research (NIHR) | *Initial survey* Patients/carers: n=341 (78%) Health professionals: n=132 (27%) Unspecified: n=20 (4%)  *Interim survey* Patients/carers: n=399 (78%) Health professionals: n=106 (21%) Unspecified: n=9 (1%)  *Workshop* Patients: n=11 (28%) Health professionals: n=17 (42%) Researchers: n=7 (18%) Facilitators: n=4 (10%) Observer: n=1 (2%) | Initial: 52 Final: 14 |
| Fackrell et al.^79^ | November 2012 | hyperacusis ^b^ | UK | National Institute of Health Research Nottingham Biomedical Research Centre*^a^;  The British Society of Audiology and Action on Hearing Loss | *Initial survey* Children/adolescents/people with lived experience: n=181 (58%) Parents/Caregivers/Family members: n=22 (7%) Clinicians/Healthcare professionals: n=87 (28%) Educational professionals: n=3 (1%) Family/friends: n=3 (4%) Not specified: n=17 (5%)  *Interim survey* Children/adolescents/people with lived experience: n=218 (67%) Parents/Caregivers/Family members: n=18 (6%) Clinicians/Healthcare professionals: n=82 (25%) Educational professionals: n=2 (1%) Family/friends: n=4 (1%) Not specified: n=3 (1%)  *Workshop* Children/adolescents/people with lived experience: n=6 (29%) Parents/Caregivers/Family members: n=5 (24%) Clinicians/Healthcare professionals: n=10 (48%) | Initial:2370 Final: 10 |
| Buckley et al.^80^ | June 2010 | Urinary incontinence^b^ | UK | National University of Ireland*^a^;  Cochrane Collaboration’s Prioritisation Fund;  James Lind Alliance | Unspecified | Initial: 226 Final: 10 |
| Elwyn et al.^81^ | June 2010 | Asthma ^b^ | UK | Department of Primary Care and Public Health School of Medicine, Cardiff University*^a^;  Asthma UK*;  British Thoracic Society* | *Initial survey: n=370* Children/adolescents/people with lived experience Parents/Caregivers/Family members  *Interim survey* Clinicians/Healthcare professionals  *Workshop* Children/adolescents/people with lived experience: n=9 (53%) Clinicians/Healthcare professionals: n=4 (23%) Researchers: n=4 (23%) | Initial: 267 Final: 10 |

^a^ PSP was done in direct partnership with JLA

^b^ Peds vs. Lifespan

^c^ More than 1 final top 10 list was generated

^d^ Participants could select more than one category

^e^ Study identified through grey literature search

^f^ No associated publication in peer reviewed journal

* Organization involved with leading the PSP
